# Machine-Learning-Guided Screening of Advantageous Solvents for Solid Polymer Electrolytes in Lithium Metal Batteries

**DOI:** 10.1021/acs.nanolett.5c00797

**Published:** 2025-05-02

**Authors:** Jiadong Shen, Junjie Chen, Xiaosa Xu, Jin Li, Zhenyu Wang, Pengzhu Lin, Zixiao Guo, Yu Wang, Jing Sun, Baoling Huang, Tianshou Zhao

**Affiliations:** † Department of Mechanical and Aerospace Engineering, The Hong Kong University of Science and Technology, Clear Water Bay, Kowloon, Hong Kong SAR 999077, China; ‡ Department of Mechanical and Energy Engineering, Southern University of Science and Technology, Shenzhen, 518055, China

**Keywords:** high-throughput DFT calculations, machine learning algorithms, solid polymer electrolytes, residual solvents, lithium metal batteries

## Abstract

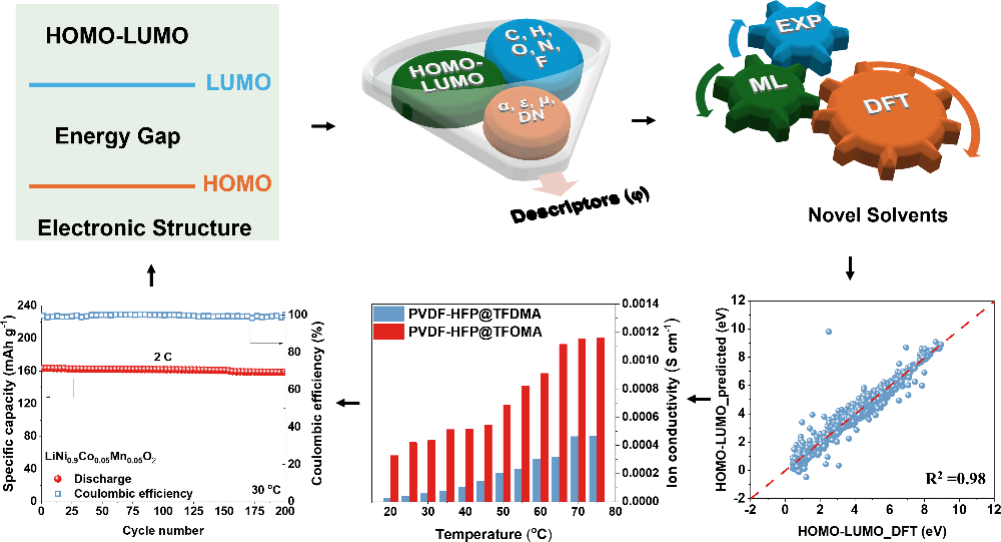

Trace residual solvents in solid polymer electrolytes (SPEs) significantly affect electrolyte and interface properties, where optimal selection enhances the ionic conductivity and transference numbers. However, the solvent complexity hinders general screening methods. We establish a universal criterion linking electronic (highest occupied molecular orbital (HOMO), lowest unoccupied molecular orbital (LUMO)) and macroscopic properties (dielectric constant, dipole moment, polarizability) via machine learning on an ∼10 000-solvent dataset from high-throughput density functional theory (DFT). Two solvents, *N*-methoxy-*N*-methyl-2,2,2-trifluoroacetamide and 2,2,2-trifluoro-*N*,*N*-dimethylacetamide were identified. Experimental incorporation of trace *N*-methoxy-*N*-methyl-2,2,2-trifluoroacetamide into a poly(vinylidene fluoride-co-hexafluoropropylene) matrix achieves a 4.5 V window, conductivity of 5.5 × 10^–4^ S cm^–1^ (30 °C), and Li^+^ transference number of 0.78. The cell retains 86.7% capacity over 500 cycles (LiFePO_4_) and 98.7% after 200 cycles at 2C (LiNi_0.9_Co_0.05_Mn_0.05_O_2_), outperforming 2,2,2-trifluoro-*N*,*N*-dimethylacetamide, dimethylformamide, *N*-methyl-2-pyrrolidone, and dimethyl sulfoxide. This synergy enables balanced ion transport, wide stability, and cycling durability, advancing safer high-energy lithium metal batteries. Our integrated approach establishes a solvent screening paradigm for rational SPE design, accelerating next-generation battery development.

The rapid expansion of electric vehicle markets has heightened demands for batteries with greater energy density and improved safety, thereby intensifying interest in solid-state lithium metal batteries.^1−3^ Inorganic solid electrolytes, such as oxides, sulfides, and halides, exhibit high ionic conductivity but suffer from significant interfacial resistance due to inadequate solid–solid contact, limiting their commercial application.^4−6^ By comparison, solid polymer electrolytes (SPEs) including poly(ethylene oxide), polydioxolane, poly(vinylidene fluoride), and particularly poly(vinylidene fluoride-co-hexafluoropropylene) (PVDF-HFP) have attracted considerable attention for their stable electrode interfaces and favorable processability.^7,8^ However, SPEs often have lower ionic conductivity, chemical stability, and mechanical strength than inorganic materials.^9,10^ Strategies such as incorporating inorganic fillers can boost mechanical rigidity,^11,12^ but introducing organic solvents remains simpler and is widely adopted^13,14^ to enhance lithium salt dissociation and stabilize interfaces.^15−21^ A typical example is the partial polymerization of dioxolane into polydioxolane,^22^ leaving residual monomers that enrich local lithium salt concentrations and improve ion transport. Likewise, solvents like *N,N*-dimethylformamide (DMF), employed to dissolve PVDF-HFP, strongly interact with polymer chains and are difficult to remove completely.^13,15^ Rather than discarding such residues as impurities, they can enhance battery performance by creating locally high salt concentrations.^23,24^ Hence, clarifying how deliberately added or inadvertently retained solvents affect the performance of SPEs is crucial. Yet, choosing suitable solvents, whether for casting or performance enhancers, remains challenging.

From a materials science perspective, the highest occupied (HOMO) and the lowest unoccupied (LUMO) molecular orbital levels of solvent molecules strongly influence their macroscopic properties, such as polarizability (α), dipole moment (μ), dielectric constant (ε), and donor number (DN),^25,26^ which dictate Li^+^ coordination and transport in solid polymer electrolytes.^27^ Leveraging high-throughput density functional theory (DFT) and machine learning, we aim to directly link these electronic descriptors to improve ion transport and interfacial stability. This integrated strategy identifies promising solvents for safer, high-performance lithium metal batteries. High polarity can facilitate salt dissociation but may aggravate lithium corrosion.^28^ Optimal ε values (25–30) help balance solvation and interfacial reactions,^26,28^ while high DN can improve Li^+^ coordination at the expense of transport.^29,30^ HOMO and LUMO dictate electrochemical stability.^27^ Unlike crystalline materials, where band structures have guided catalysts and battery electrode designs,^31−34^ solvent structure–property correlations are less established, often relying on empirical or trial-and-error solvent blending.^35−37^ This raises complexity, cost, and compatibility concerns.^38^ Machine learning algorithms have recently become powerful tools for material development,^39−43^ but face similar drawbacks as earlier high-throughput DFT calculations:^44,45^ they remain predominantly theoretical, lack experimental validation, and rely heavily on data quality, emphasizing the need to integrate experiments with theoretical models.^46−48^

Here, we screened ∼10 000 organic solvents containing C, H, O, N, and F to identify beneficial residues for SPEs. High-precision DFT calculations revealed correlations between HOMO-LUMO gaps and μ, ε, α, and DN, verifying the pivotal role of the electronic structure in shaping solvent properties. We then employed Extreme Gradient Boosting (XGBoost),^49^ Sure Independence Screening and Sparsifying Operator (SISSO),^50^ and crystal graph convolutional neural network (CGCNN)^51^ to establish a predictive model, proposing criteria for low-polarity solvents. Among these, 2,2,2-trifluoro-*N,N*-dimethylacetamide (TFDMA)^52,53^ and *N*-methoxy-*N*-methyl-2,2,2-trifluoroacetamide (TFOMA) emerged as top candidates. TFOMA’s additional methoxy group appears to enhance ionic pathways.^26,28,30^ Experimentally, trace TFOMA in PVDF-HFP delivers a wider voltage window (>4.5 V), higher ionic conductivity (5.5 × 10^–4^ S cm^–1^ at 30 °C), and a larger lithium-ion transference number (0.78), along with lower lithium plating/stripping overpotential. In LiFePO_4_ and LiNi_0.9_Co_0.05_Mn_0.05_O_2_ (NCM91) systems, PVDF-HFP@TFOMA exhibits excellent cycling stability, retaining 98.9% capacity at 2 C after 200 cycles. This study not only advances SPE research but also demonstrates a data-driven, experimentally validated paradigm for solvent design in lithium metal batteries.

## Screening and Prediction Workflow

**Figure 1:**
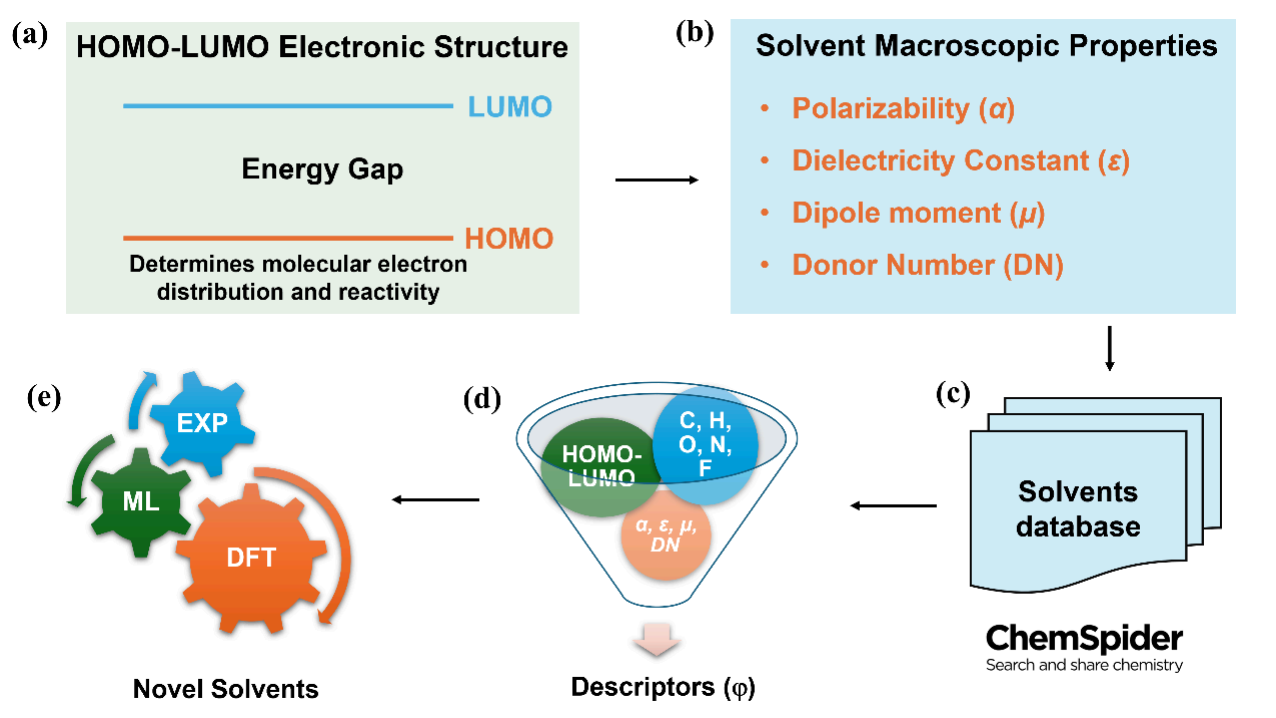
(a, b) Starting from the potential relationship between the electronic structure of organic compounds’ HOMO–LUMO (panel (a)) and corresponding solvent properties (α, ε, μ, and DN) (panel (b)). In panel (c), data extraction was performed by connecting with the Chemical Spider database. In panel (d), elements were restricted to C, H, O, N, and F to build relationships between HOMO–LUMO, and macroscopic solvent properties, leading to the development of descriptors. (e) Based on DFT results, a machine learning model was trained to predict solvents, followed by experimental validation of the predicted solvents.

Molecular orbital energy levels (HOMO and LUMO) are key parameters for characterizing the electronic structures of solvent molecules, while properties such as α, ε, μ, and DN reflect the macroscopic physicochemical properties of solvents.^26^ Based on the fundamental principle that electronic structure determines macroscopic properties, we designed a systematic screening strategy for solvent molecules. As shown in Figure 1, we extracted organic solvent molecules containing C, H, O, N, and F elements from the ChemSpider database^54^ and obtained basic physical parameters, including HOMO and LUMO energy levels, α, and μ, through high-throughput DFT calculations. The ε parameter was calculated using the Clausius–Mossotti equation,^55^ and the DN parameter was determined by calculating the interaction energies between solvent molecules and the Lewis acid SbCl_5_.^29,56^ We first established structure–property relationships between molecular orbital energy levels (HOMO−LUMO gap) and ε as well as μ. On this basis, further DFT calculations were conducted to explore the interfacial properties of solvents, including *DN* and Li binding energies. Subsequently, based on the dataset obtained from high-throughput calculations, we employed machine learning algorithms to deeply reveal the intrinsic connections between electronic structures and macroscopic properties, and experimentally validated the relevant predictions. The detailed screening and prediction process is illustrated in Figure S1.

## High-Throughput DFT Calculations

**Figure 2:**
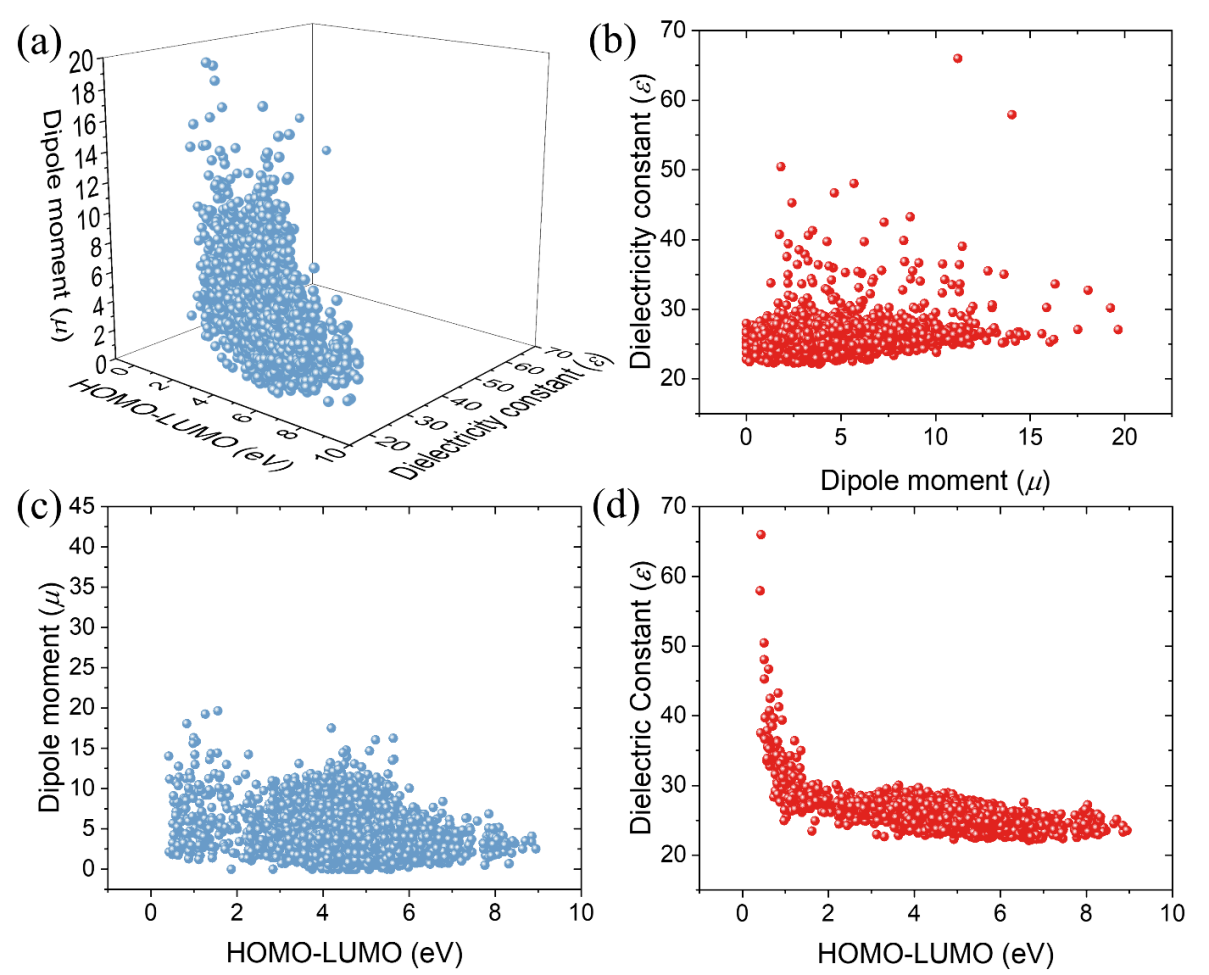
(a) 3D scatter plot showing relationships among the HOMO–LUMO gap, dipole moment (μ), and dielectric constant (ε); (b) dipole moment (μ) vs dielectric constant (ε); (c) dipole moment (μ) vs. HOMO-LUMO gap; (d) dielectric constant (ε) vs HOMO–LUMO gap.

Building upon our systematic screening strategy, we analyzed the relationships among the HOMO–LUMO gaps, μ, and ε of the solvent molecules obtained from high-throughput DFT calculations. These relationships are presented in Figure 2. Additionally, the correlations between the relative polarizabilities of the molecules and the solvents’ ε value, μ value, and HOMO–LUMO gaps are depicted in Figure S2. As shown in Figure 2a, the HOMO–LUMO gaps of the solvent molecules are primarily concentrated between 2 eV and 6 eV, in the μ range from 0 to 15 Debye (D), and the ε values are predominantly between 22 and 30. Figure 2b illustrates a significant positive correlation (slope ≈ 0.11) between the μ and ε values of the solvent molecules, indicating that molecules with larger μ values tend to exhibit higher ε values. Similar positive correlations between both isotropic and anisotropic relative α and ε values (Figures S2a and S2b) are revealed in further analyses. This fundamental relationship stems from the contributions of deformation polarizability and orientation polarizability to the dielectric constant (Supplementary Note 1), where the Clausius–Mosotti equation and Debye’s theory jointly rationalize how μ and α determine ε. This correlation is also evident in the relationships between relative polarizability and μ (Figures S2c and S2d). These findings suggest a universal correlation between the microscopic structural characteristics of solvent molecules and their macroscopic dielectric properties: larger μ values and relative polarizabilities generally correspond to higher ε.

Moreover, we investigated how the μ and ε values of the molecules vary with their electronic structures, as shown in Figures 2c and 2d. It can be observed that, within the HOMO–LUMO gap range of 0.5–2 eV, the μ and ε values of the solvents decrease sharply as the gap increases. This trend becomes significantly less pronounced when the gap further increases to the range of 2–8.5 eV. Such a nonlinear dependence on the HOMO–LUMO gap can be understood via second-order perturbation theory, wherein polarizability exhibits a strong inverse correlation with the gap (Supplementary Note 2), thus explaining the sharp decline in μ and ε when the gap is small. As the gap increases, the charge distribution within the molecule becomes more localized, leading to a gradual decrease in polarity.^57,58^ A similar phenomenon is observed in the polarizabilities of the molecules, as shown in Figures S2e and S2f. These insights, derived from correlating various physical properties of solvent molecules, provide a new perspective for understanding the relationship between the electronic structure and the properties of solvents. These correlations emphasize that tuning HOMO/LUMO energies, dipole moment, and dielectric constant is essential for balancing Li^+^ solvation and chemical stability in polymer electrolytes. Such an understanding offers important guidance for the rational design of electrolyte solvents in lithium-ion batteries.

## Construction and Validation of the Machine Learning Model

To develop our predictive framework, we first assembled a dataset of solvents and their key properties (including dielectric constant (ε), dipole moment (μ), and HOMO–LUMO energy levels) from the ChemSpider database,^54^ a resource that provides verified chemical structures and physicochemical data. By leveraging ChemSpider, we ensured comprehensive coverage of diverse solvents to support subsequent property calculations and feature extraction. Building on this foundation, we then employed a multilevel machine learning strategy encompassing XGBoost,^49^ SISSO,^50^ and CGCNN,^14^ aiming to balance interpretability and predictive accuracy. Specifically, XGBoost and SISSO were selected because they provide a relatively straightforward interpretation of feature importance, making them well-suited for quickly screening large numbers of descriptors. Meanwhile, CGCNN excels at capturing complex nonlinear interactions, particularly for periodic structures, thus extending predictive capabilities to systems where local atomic environments significantly influence solvent properties. Initially, we used interpretable algorithms such as XGBoost^49^ and SISSO^50^ as screening tools to identify critical features and assess predictive performance. As illustrated in Figure S3a, neglecting HOMO–LUMO descriptors resulted in poor accuracy for predicting ε (*R*^2^ = 0.19). Incorporating these orbital-level descriptors significantly enhanced the model (*R*^2^ = 0.60, Figure S3b), with feature importance analysis (Figure S3c) revealing that electronic structure attributes contributed over 65%. SISSO corroborated these findings: using the HOMO−LUMO gap alone yielded *R*^2^ = 0.65 (Figure S3d), while additional parameters (molecular weight, van der Waals volume) offered minimal improvement (Δ*R*^2^ < 0.02, Figures S3e and S3f). Conversely, models relying purely on geometric features (e.g., bond lengths, dihedral angles) performed poorly (*R*^2^ < 0.1, Figure S3g), underscoring the decisive role of electronic structure in shaping solvent properties.

**Figure 3:**
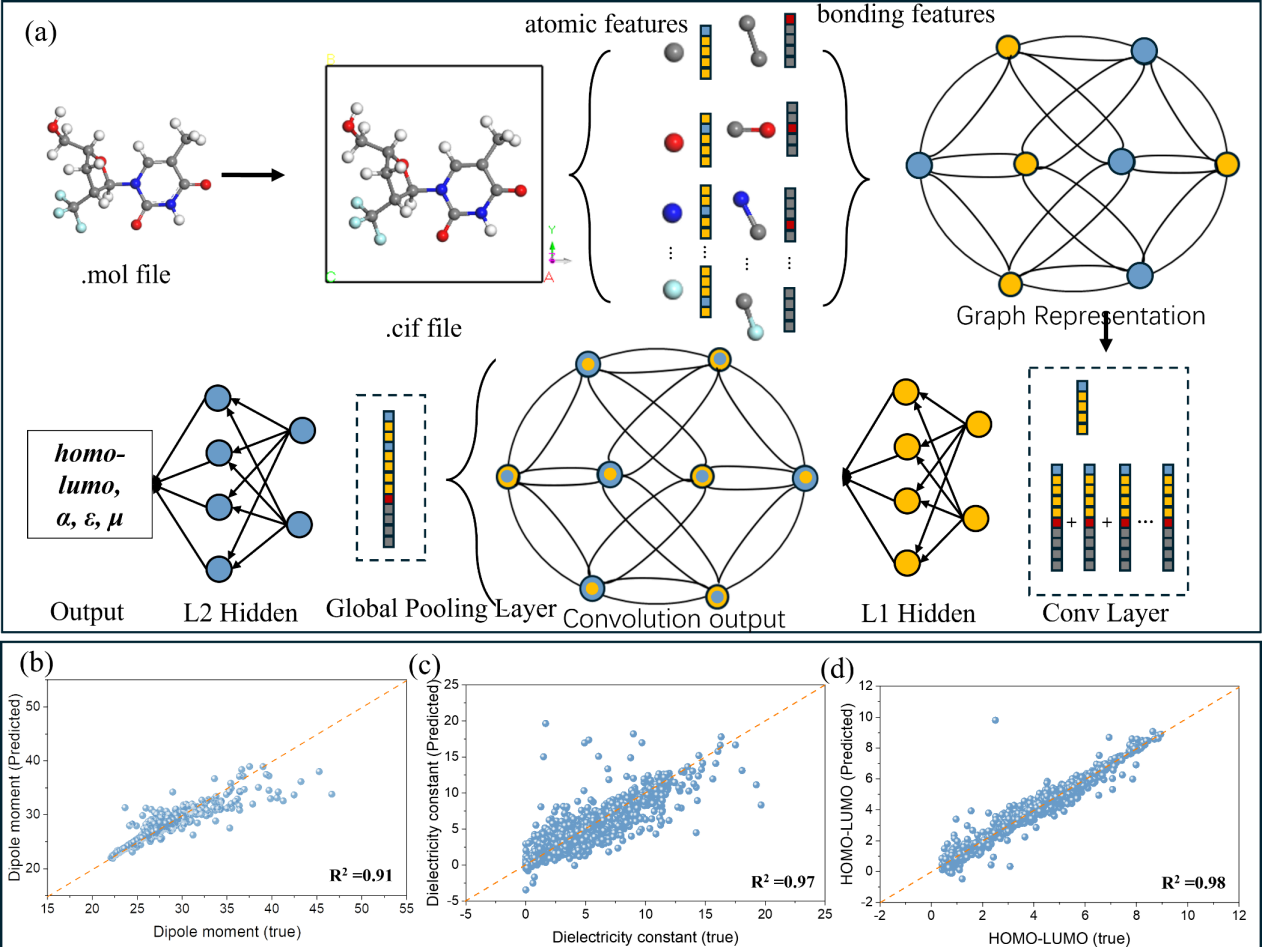
(a) Workflow of the CGCNN model construction. (b–d) Prediction results on the test dataset for (b) dielectric constant (ε), (c) dipole moment (μ), and (d) HOMO−LUMO.

Despite these advances, highly nonlinear properties such as μ remained challenging for XGBoost and SISSO (Figures S3h and S3i). To address this limitation, we implemented the CGCNN^51^ algorithm, originally designed for crystalline materials, by representing each solvent molecule under periodic boundary conditions (see Figure 3a and Figure S4). In this approach, atoms and bonds become nodes and edges in a graph network, enabling the capture of both intramolecular and intermolecular interactions. Through multiple convolutional, hidden, and pooling layers, CGCNN automatically learns hierarchical structural and electronic features. Remarkably, it achieved *R*^2^ = 0.91 for μ, 0.97 for ε, and 0.98 for the HOMO–LUMO gap (Figures 3b–d). To further explore the “black box” nature of deep learning, we performed dimensionality reduction analyses, revealing strong correlations between the CGCNN’s latent embeddings and electronic-structure descriptors (Table S1). Notably, the predicted outputs shifted from strong alignment with LUMO levels in one subset (*r* = 0.937, *p* < 10^–30^) to closer correspondence with HOMO energies in another (*r* = –0.568, *p* < 10^–30^), illustrating the model’s adaptability to distinct quantum-chemical contexts. Further principal component and *t*-SNE evaluations showed stable correlations between latent coordinates and dipole moments (e.g., PC1 vs dipole: *r* = –0.506, *p* < 10^–30^), indicating that these internal representations capture crucial molecular orbital and electron density features. The SHAP contribution analysis (Figures S5 and S7) clarifies how each descriptor influences the predicted properties. Specifically, Figures S7a and S7b, which were derived from the XGBoost models, highlight the dominant role of frontier orbitals (HOMO, LUMO) in determining both the dielectric constant and the dipole moment. This finding is consistent with the known importance of electronic structure in polarization response and charge redistribution. The positive SHAP values for higher-energy HOMOs suggest stronger dielectric effects through easier electron donation, while lower LUMO energies enhance molecular polarizability. In addition, total polarizability and electronegative-atom fractions further underscore the impact of molecular asymmetry on dipole moments. In contrast, Figures S7c and S7d, which were obtained from the CGCNN models, show that latent embeddings rank highly for the HOMO–LUMO gap, capturing deeper structural–electronic correlations beyond simpler atomic descriptors. Their consistent relevance confirms alignment with fundamental quantum-chemical factors and reinforces model interpretability. Overall, these results demonstrate that the chosen descriptors, along with the latent GNN features, accurately mirror known theoretical dependencies on orbital energies and polarizability, thereby lending confidence to the robustness and transparency of our approach. Further confirming its robustness, we also carried out 50-fold cross-validation, multiple-seed tests, and *y*-scrambling (Figure S8), which demonstrated stable prediction accuracy and supported genuine structure–property learning rather than mere data memorization. Overall, this integrated approach provides a solid theoretical basis for applying deep neural networks in molecular design linking electronic structures to macroscopic solvent properties.

## Establishing and Applying Solvent Screening Criteria for Novel Solvents

**Figure 4:**
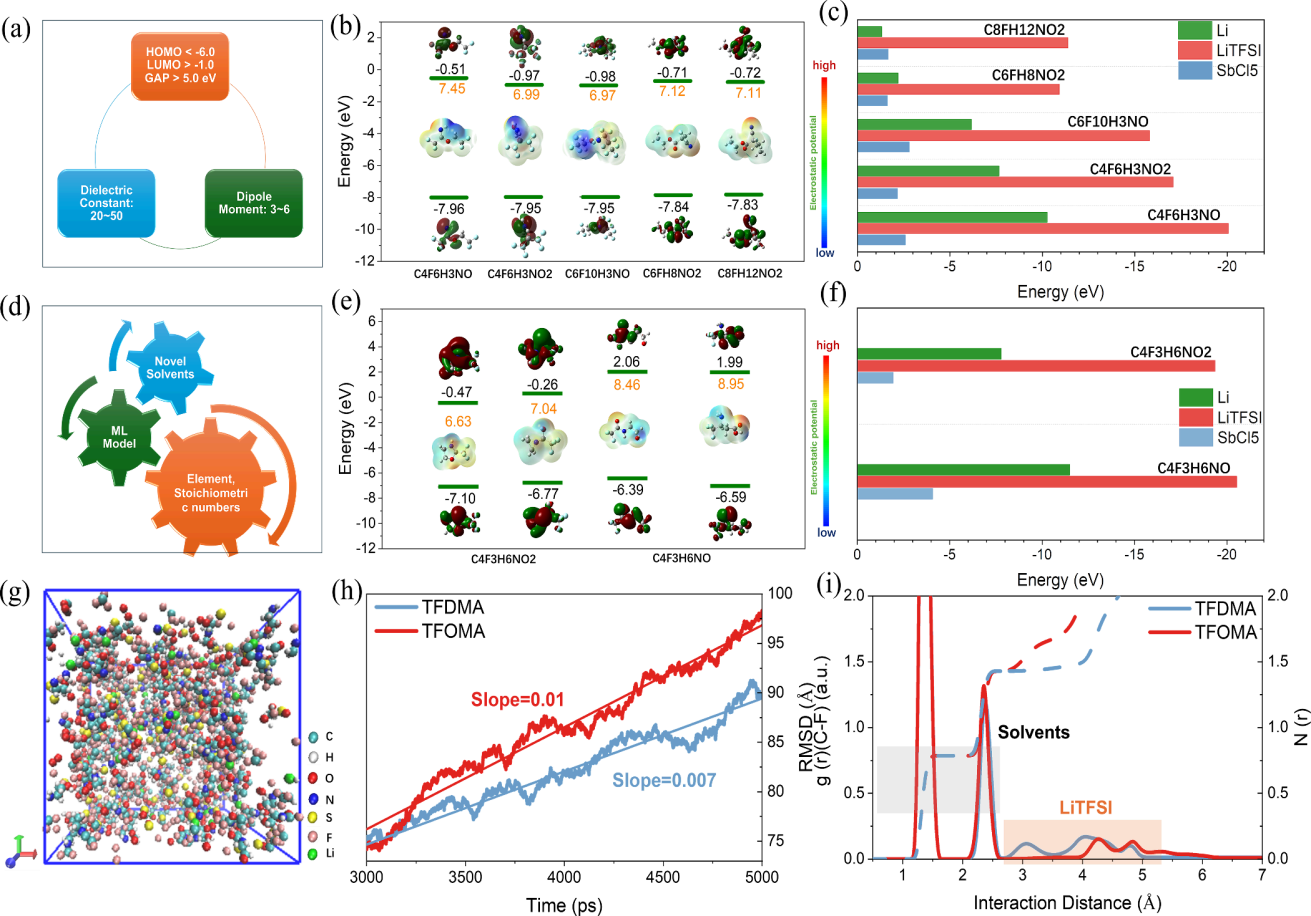
(a) Solvent screening criteria; (b) HOMO–LUMO results and molecular structures for the top 5 candidate solvents meeting the criteria; (c) binding energies of the top five solvents with typical lithium salt (LiTFSI) and a representative Lewis acid (SbCl_5_), including Li binding energy; (d) prediction of novel solvent properties using element-based features and trained machine learning models; (e) HOMO−LUMO results and molecular structures of two predicted novel solvents; (f) binding energies between the predicted solvents and typical LiTFSI and SbCl_5_. (g) Schematic of the TFOMA solvent with a local high concentrated LiTFSI salt model; (h) 5 ns RMSD curves of lithium ions in TFDMA and TFOMA in an equilibrated system from molecular dynamic simulation; (i) radial distribution functions (RDFs) of C–F atomic pairs associated with LiTFSI and solvent molecules in TFOMA and TFDMA.

Building on our systematic solvent analysis (Table S2), we defined quantitative criteria (Figure 4a) to identify ideal candidates: HOMO < –6 eV, LUMO > –1 eV, a HOMO–LUMO gap > 5 eV, ε values in the 20–50 range, and μ values between 3 D and 6 D. Such parameters balance oxidative/reductive stability (via HOMO and LUMO), sufficient salt dissociation (ε), and moderated ion–solvent interactions (μ).^27,29,37^ Through statistical ranking of molecules that met these benchmarks, the top 10 solvents were identified, with five leading contenders (C_4_F_6_H_3_NO, C_4_F_6_H_3_NO_2_, C_6_F_10_H_3_NO, C_6_FH_8_NO_2_, C_8_FH_12_NO_2_) shown in Figure 4b (the remaining five are shown in Figure S9). Their interactions with LiTFSI, SbCl_5_, and Li were examined (see Figure 4c, as well as Figures S10–S12), using SbCl_5_ adsorption energies to quantify DN.^29^ As illustrated in Figure S13, DN positively correlates with the HOMO–LUMO gap, indicating stronger electron donation for wider gaps. However, solvents with higher DN often exhibit weaker Li^+^ acceptance, reflecting a tradeoff in coordinating Li ions.

Expanding on this insight (Figure 4d), we identified two promising, previously untrained solvents: 2,2,2-trifluoro-*N,N*-dimethylacetamide (TFDMA) (C_4_H_6_F_3_NO) and *N*-methoxy-*N*-methyl-2,2,2-trifluoroacetamide (TFOMA) (C_4_H_6_F_3_NO_2_) (Figure 4e). TFDMA has been experimentally validated,^48^ while TFOMA is proposed in this study for the first time. TFOMA’s HOMO (–7.10, –6.77 eV) and LUMO (–0.47, –0.26 eV) suggest enhanced high-voltage stability and favorable SEI formation. By contrast, TFDMA’s apparently positive LUMO (2.06, 1.99 eV) may reflect computational parameter differences and thus warrants caution. MD simulations further support TFOMA’s superior interfacial behavior and ionic conductivity (see Figure 4f, as well as Tables S3 and S4). In Figure 4g, TFOMA exhibits localized high-concentration structures with lithium salts, while the Li^+^ root mean square displacement (Figure 4h) is greater for TFOMA (0.01 Å ns^–1^) than TFDMA (0.007 Å ns^–1^). Additional simulations at 300–340 K (Figures S14a–S14d) confirm accelerated Li^+^ transport. The radial distribution function (Figure 4i) reveals longer C–F distances and more concentrated distributions in TFOMA, implying a distinct solvation mechanism (also see Figures S14e and S14f). We attribute this to the extra methoxy group in TFOMA, which forms new ion channels, thereby boosting the macroscopic conductivity. This emphasizes the importance of functional group engineering for optimizing electrolyte performance.

## Electrochemical Performance Testing and Mechanistic Analysis of Solid-State Lithium Metal Full Cells

**Figure 5:**
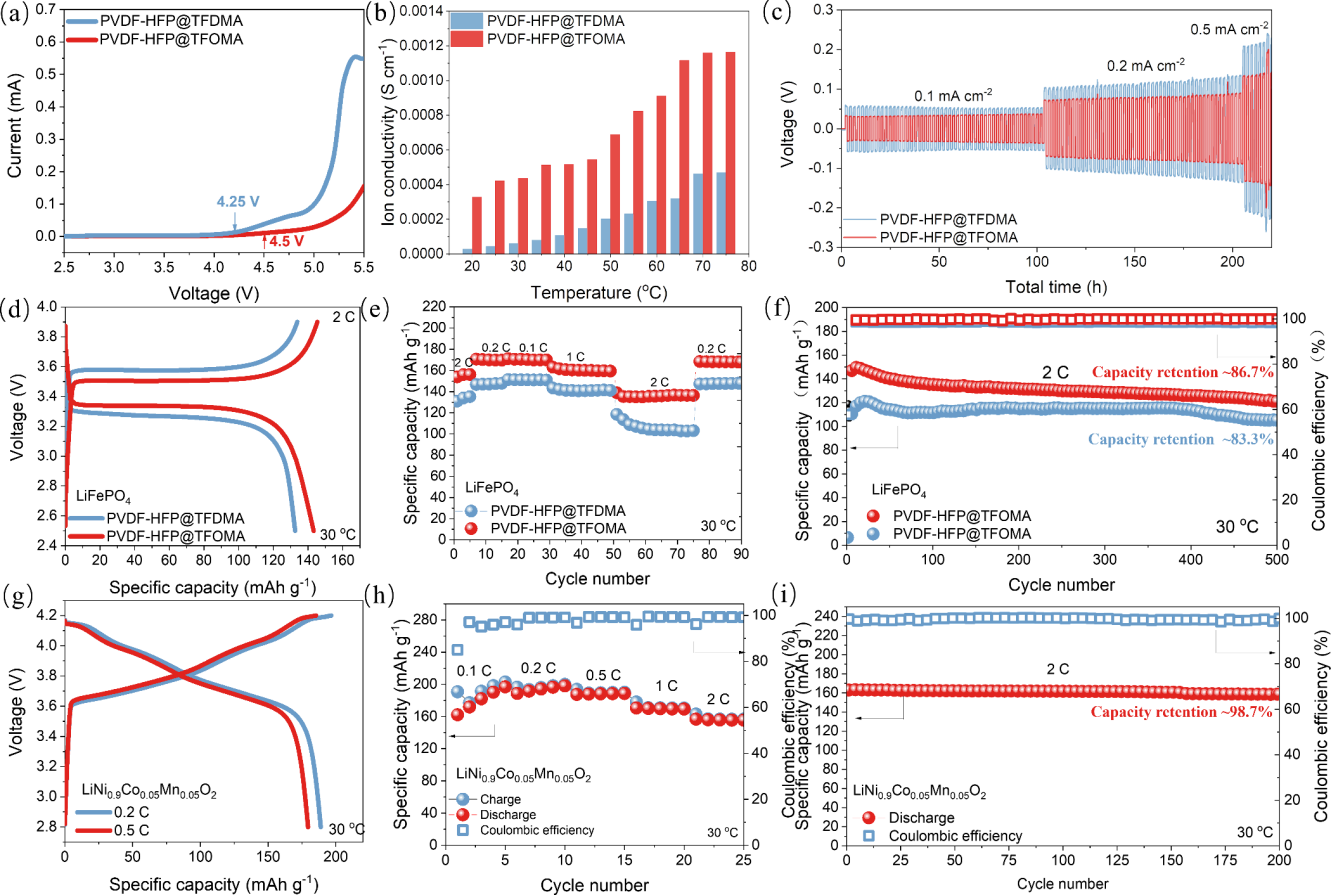
(a–c) Comparison of LSV test curves (panel (a)), lithium-ion conductivity results (panel (b)), and deposition/stripping electrochemical performance (panel (c)) between PVDF-HFP@TFDMA and PVDF-HFP@TFOMA solid electrolytes. (d–f) Charge–discharge curves (panel (d)), rate performance (panel (e)), and long-cycle stability at 2 C (1 C = 175 mAh g^–1^) (panel (f)) of LiFePO_4_ cathode with PVDF-HFP@TFDMA and PVDF-HFP@TFOMA solid electrolytes. (g–i) Charge–discharge curve (panel (g)), rate performance (panel (h)), and electrochemical performance at 2 C (1 C = 223 mAh g^-1^) (panel (i)) of LiNi_0.9_Co_0.05_Mn_0.05_O_2_ cathode with PVDF-HFP@TFOMA solid electrolyte.

To validate our design paradigm, we fabricated PVDF-HFP-based solid polymer electrolytes (SPEs) with TFOMA or TFDMA as the residual solvent. PVDF-HFP was selected for its excellent high-voltage stability and strong F**···**F interactions with fluorinated solvents,^19,48^ which promote a uniform solvent distribution and retain beneficial traces of solvent for enhanced lithium-ion conduction. Elemental mapping confirmed a uniform distribution of C, N, O, F, and S in membranes ∼23 μm thick (see Figures S15 and S16). A trace (∼0.1 μL) of each solvent was introduced during cell assembly to ensure a consistent residual content. Linear sweep voltammetry (Figure 5a) reveals that PVDF-HFP@TFOMA can withstand oxidation up to 4.5 V, whereas PVDF-HFP@TFDMA oxidizes at 4.25 V. The improved oxidative stability in TFOMA-based electrolytes is attributed to TFOMA’s lower HOMO energy and reduced polarity, which collectively suppress unwanted cathode–solvent interactions. At 30 °C, ionic conductivity measurements (Figure 5b) show a marked difference: 5.5 × 10^–4^ S cm^–1^ for TFOMA versus 1.0 × 10^–4^ S cm^–1^ for TFDMA. Above 70 °C, the TFOMA system exceeds 10^–3^ S cm^–1^, underscoring its superior ion transport properties. Arrhenius fits yield activation energies of 0.29 eV (TFOMA) and 0.45 eV (TFDMA) (Figure S17), indicating more efficient Li^+^ migration with TFOMA. The methoxy moiety in TFOMA likely creates additional ion coordination sites, reducing ion–anion coulombic interactions, and fostering higher lithium-ion transference (0.78 vs 0.75; see Figure S18).

In lithium metal symmetric cells at 0.1–0.5 mA cm^–2^, the TFOMA-based electrolyte achieves plating/stripping overpotentials of ±0.025–0.08 V, significantly lower than ±0.05–0.19 V for TFDMA (Figure 5c). When paired with a LiFePO_4_ cathode (2 C at 30 °C), PVDF-HFP@TFOMA delivers 144 mAh g^–1^ versus PVDF-HFP@TFDMA’s 130 mAh g^–1^ (Figure 5d). Subsequent rate tests yield capacities of 172, 170, 162, 144, and 170 mAh g^–1^ upon cycling at 0.1, 0.2, 1, 2, and back to 0.2 C, respectively (Figure 5e). Over 500 cycles at 2 C, capacity retention remains 86.7%, exceeding TFDMA (83.3%) with a Coulombic efficiency near 99.1% (see Figures 5f, as well as Figure S19). Furthermore, under a high-voltage LiNi_0.9_Co_0.05_Mn_0.05_O_2_ (NCM91) cathode, TFOMA demonstrates even better performance, achieving 198 and 175 mAh g^–1^ at 0.2 and 0.5 C (Figure 5g) and retaining 98.7% of its capacity after 200 cycles at 2 C (Figure 5i). Such robust cycling confirms the practical advantage of TFOMA in high-voltage settings, as summarized in Table S5, highlighting our data-driven solvent design strategy for next-generation lithium metal batteries. Meanwhile, Table S6 shows that previous ML-based solvent-screening studies typically focused on narrower property sets with limited validation, whereas our integrated approach broadens predictive coverage and offers deeper experimental verification for advanced SPE development.

In conclusion, we developed a comprehensive strategy for designing and screening solvents for SPEs in lithium metal batteries. High-throughput DFT calculations covering nearly 10 000 candidates uncovered key correlations between HOMO and LUMO levels and macroscopic properties (α, μ, ε, DN). Using machine learning (XGBoost, SISSO, and CGCNN), we built predictive models, established universal screening criteria, and identified five promising solvents. Among them, the newly proposed TFOMA, with its lower polarity and reduced Li-ion interactions, outperformed PVDF-HFP@TFDMA with a 4.5 V electrochemical window, an ionic conductivity of 5.5 × 10^–4^ S cm^–1^ (30 °C), and enhanced Li-metal stability. Cells with PVDF-HFP@TFOMA retained 86.7% of their capacity after 500 cycles using LiFePO_4_ and 98.7% after 200 cycles at 2 C with NCM91. Our integrated approach merges first-principles theory, machine learning, and experimental validation, accelerating materials discovery and deepening our understanding of solvents’ electronic structure–property relationships. This data-driven method can be extended to other advanced electrolyte designs for high-performance, safe, solid-state lithium metal batteries.

## Data Availability

All code and models are publicly available at: https://github.com/mejiadongs. Training datasets can be requested from the authors with valid research justification.

## Supporting Information Available

Detailed high-throughput calculation protocols, machine learning model construction and validation procedures, experimental methods, electrochemical testing and characterization data, and additional figures and tables (PDF)
