# Supplementary material for: Machine-Learning-Guided Screening of Advantageous Solvents for Solid Polymer Electrolytes in Lithium Metal Batteries
Source: Nano Lett. 2025 May 2;25(19):7801–9. doi: 10.1021/acs.nanolett.5c00797 (PMC12082693; doi:10.1021/acs.nanolett.5c00797)
Supplement: Supplementary file 1 — nl5c00797_si_001.pdf [file nl5c00797_si_001.pdf]

Supplementary Information for

Machine Learning-Guided Screening of Advantageous Solvents for Solid Polymer Electrolytes in Lithium Metal Batteries

Jiadong Shen[1], Junjie Chen[1], Xiaosa Xu[1], Jin Li[1], Zhenyu Wang[1], Pengzhu Lin[1], Zixiao Guo[1], Yu Wang[1], Jing Sun[1], Baoling Huang[1*], Tianshou Zhao[1,2*]*

J. Shen, J. Chen, X. Xu, J. Li, Z. Wang, P. Lin, Z. Guo, Y. Wang, J. Sun, B.L. Huang, T.S. Zhao

[1] Department of Mechanical and Aerospace Engineering, The Hong Kong University of Science and Technology, Clear Water Bay, Kowloon, Hong Kong SAR, China

E-mail: jsunav@connect.ust.hk (J. Sun); mebhuang@ust.hk (B.L. Huang).

[2] Department of Mechanical and Energy Engineering, Southern University of Science and Technology, Shenzhen, 518055, China

E-mail: zhaots@sustech.edu.cn (T.S. Zhao)

Supplementary note:

(1) Theoretical Framework for Dielectric Constant Calculation from Molecular Properties[1]

The relationship between molecular properties and dielectric constants can be established through the Clausius-Mosotti equation, which correlates the dielectric constant with molecular polarizability:

$$\frac{\varepsilon_r - 1}{\varepsilon_r + 2} = \frac{N\alpha}{3\varepsilon_0}$$

where ε_r represents the relative dielectric constant, N denotes the number density of molecules, α is the total molecular polarizability, and ε_0 is the vacuum permittivity. By expressing the number density in terms of Avogadro's constant N_A and molar volume V_m:

$$N = \frac{N_A}{V_m}$$

The Clausius-Mosotti equation can be rearranged to directly solve for the dielectric constant:

$$\varepsilon_r = \frac{1 + 2\frac{N_A\alpha}{3\varepsilon_0 V_m}}{1 - \frac{N_A\alpha}{3\varepsilon_0 V_m}}$$

For molecules possessing permanent dipole moments, the total molecular polarizability α comprises two primary contributions: (1) Deformation polarizability α_d (encompassing both electronic and atomic polarizability); (2) Orientation polarizability α_o (arising from permanent dipole moments). According to Debye's theory, the orientation polarizability is expressed as:

$$\alpha_o = \frac{\mu^2}{3k_B T}$$

Consequently, the total molecular polarizability can be written as:

$$\alpha = \alpha_d + \frac{\mu^2}{3k_B T}$$

Substituting this expression into the previous equation yields the comprehensive relationship between the dielectric constant and molecular properties:

$$\varepsilon_r = \frac{1 + 2\frac{N_A}{3\varepsilon_0 V_m}\left(\alpha_d + \frac{\mu^2}{3k_B T}\right)}{1 - \frac{N_A}{3\varepsilon_0 V_m}\left(\alpha_d + \frac{\mu^2}{3k_B T}\right)}$$

where: N_A is Avogadro's constant (6.02214076×10^{23} mol^{-1}); ε_0 is the vacuum permittivity ($8.8541878128\times10^{-12}$ F/m); k_B is the Boltzmann constant (1.380649×10^{-23} J/K); α_d is the deformation polarizability; μ is the permanent dipole moment; T is the temperature; V_m is the molar volume.

(2) Relationship Between Electronic Properties and HOMO-LUMO Gap[2]

The molecular polarizability exhibits a strong inverse relationship with the HOMO-LUMO gap, which can be understood through second-order perturbation theory. The polarizability α can be expressed as:

$$\alpha \propto \sum_{n\neq 0} \frac{|\langle n|\mu|0\rangle|^2}{E_n - E_0}$$

where the dominant contribution comes from the HOMO-LUMO transition:

$$\alpha \propto \frac{|\langle LUMO|\mu|HOMO\rangle|^2}{E_{LUMO} - E_{HOMO}}$$

This relationship directly influences the dielectric constant ε through the Clausius-Mossotti relation:

$$\varepsilon = 1 + 4\pi N\alpha$$

where N represents the molecular number density. Consequently, molecules with smaller HOMO-LUMO gaps typically exhibit enhanced polarizability and higher dielectric constants, leading to stronger responses to external electric fields. The permanent dipole moment μ exhibits a more complex relationship with the HOMO-LUMO structure. In the molecular orbital framework, the dipole moment

can be expressed as a sum over all occupied molecular orbitals:

$$\vec{\mu} = -e \int \psi^* \vec{r}\, \psi d\tau = -e \sum_i^{occ} \sum_j^{all} c_i^* c_j \int \phi_i^* \vec{r}\, \phi_j d\tau$$

Where the contribution from HOMO-LUMO interaction can be explicitly written as:

$$\vec{\mu}_{HL} = -e\left(c_{HOMO^*} c_{LUMO} \int \phi_{HOMO}^* \vec{r}\, \phi_{LUMO} d\tau + c.c.\right)$$

The mixing coefficients c_i are related to the HOMO-LUMO gap through configuration interaction:

$$c_{LUMO} \approx \frac{\left\langle HOMO \middle| \hat{H'} \middle| LUMO \right\rangle}{E_{LUMO} - E_{HOMO}}$$

where ψ represents the molecular waveform. The HOMO-LUMO gap influences the dipole moment through multiple mechanisms: (1) Direct orbital distribution effects: The spatial characteristics and symmetry of the HOMO significantly influence charge density distribution and, consequently, the dipole moment magnitude; (2) Configuration mixing: Smaller HOMO-LUMO gaps facilitate greater mixing between ground and excited states, potentially breaking charge distribution symmetry and modifying the permanent dipole moment; (3) Structural effects: HOMO-LUMO interactions can influence molecular equilibrium geometry, indirectly affecting the dipole moment through conformational changes; (4) Chemical bonding: The HOMO-LUMO gap modulates bond polarization, contributing to the overall molecular dipole moment. The magnitude of the permanent dipole moment is fundamentally dependent on the extent of molecular orbital hybridization. When the HOMO-LUMO energy gap decreases, there is an increased probability of orbital mixing through configuration interaction, which may lead to an enhancement of the permanent dipole moment due to asymmetric charge distribution.

Methods:

Density Functional Theory (DFT) Calculations

High-throughput DFT calculations were performed using the Gaussian16[3] software package, with batch processing and calculations of the models carried out using ASE (Atomic Simulation Environment)[4] scripts. Approximately 10,000 molecular models were obtained from the open-source database ChemSpider for these calculations.[5] To ensure the accuracy and feasibility of our training data, we applied the following data screening and cleaning procedures: Elemental Composition,

Molecular Weight, and Functional Groups: (1) Molecules were restricted to those containing only carbon (C), hydrogen (H), oxygen (O), nitrogen (N), and fluorine (F), with molecular weights ranging from 77.06 to 1651.81 g/mol (average 349.87 g/mol). In addition, only molecules bearing one or more of the following functional groups were retained: alcohol, aldehyde, alkene, alkyne, amide, amine, ester, ether, ketone, or nitro. (2) Molecules identified as highly toxic or those that are challenging to synthesize on a large scale were excluded to prioritize safety and practical applicability. (3) Duplicate molecular entries were removed, and the structural formats were standardized to ensure consistency across the dataset. These steps balance the dataset size with practical considerations such as safety, ease of synthesis, and overall suitability for potential electrolyte applications. The geometries were optimized at the B3LYP/3-21G* level of theory,[6] and frequency calculations were conducted to confirm that the optimized structures correspond to true minima on the potential energy surface. Grimme's D3 dispersion corrections were applied to account for van der Waals interactions[7].

The adsorption energy (E_{ads}) of LiTFSI on the surface was calculated using the following formula:

$$E_{ads} = E_{total} - (E_{surface} + E_{LiTFSI})$$

where E_{total} is the total energy of the system with LiTFSI adsorbed on the surface, $E_{surface}$ is the energy of the clean surface, and E_{LiTFSI} is the energy of the isolated LiTFSI molecule. The dissociation energy (E_{diss}) of the lithium atom from the surface was calculated using:

$$E_{diss} = E_{Li/surface} - E_{surface} - E_{Li}.$$

where $E_{Li/surface}$ is the energy of the surface with the lithium atom adsorbed, $E_{surface}$ is the energy of the clean surface adsorbed with TFSI-H (Introduction of pseudo hydrogen to offset the non-neutrality of TFSI), and E_{Li} is the energy of the isolated lithium atom.

Molecular Dynamics (MD) Simulations

MD simulations were conducted using the Forcite module in Materials Studio 2019 software. Models were constructed for TFDMA, TFOMA and LiTFSI with molecular ratios consistent with experimental molar ratios. The initial MD models were built using the Amorphous Cell module in Materials Studio[8]. The force field used for the simulations was the COMPASS force field[9], and atomic charges were assigned using the force field's default parameters. The MD simulations followed standard procedures: (1) energy minimization was performed to remove any bad contacts or high-energy configurations in

the initial structure; (2) initial pre-equilibration was carried out under the NVT ensemble at 350 K for 5 ns using the Nosé-Hoover thermostat for temperature control[10]; (3) the system was then annealed by cooling to 300 K and equilibrated under the NVT ensemble for an additional 5 ns; (4) pressure equilibration was conducted under the NPT ensemble using the Berendsen barostat[11], gradually decreasing the pressure to 1 bar; (5) finally, the system was run for 15 ns at 1 bar, and data were extracted for further analysis. Trajectory visualization and analysis, including root-mean-square deviation (RMSD) calculations and radial distribution functions (RDF), were performed using the Materials Studio Analysis tools.

Machine Learning for Building Predictive Models of Novel Solvents

The machine learning (ML) component of this study involved the application of XGBoost[12], SISSO [13], and CGCNN[14] algorithms to model and predict the properties of novel solvents. A dataset comprising approximately 10,000 samples was used, sourced from the open-source database ChemSpider. To enhance the robustness and generalizability of the models, the dataset was split into training, validation, and test sets in a ratio of 6:2:2; 50-fold cross-validation was employed throughout the training process to test the robustness and reliable predictive capabilities of the model. For XGBoost and SISSO models, feature engineering involved calculating descriptors like dihedral angles, bond lengths, and electronic properties. All features were normalized to ensure they contribute equally to the model training. In addition, to enhance reproducibility and reliability of the predictions, the XGBoost model was explicitly configured with a learning rate of 0.1, 100 estimators, and a maximum tree depth of 3. Moreover, to address potential over-reliance on point estimates, an uncertainty quantification approach based on bootstrap aggregation was implemented, whereby multiple XGBoost models were trained on resampled subsets of the training data to derive confidence intervals for the predictions. For the CGCNN model, molecular models were converted into periodic crystal structures using appropriate crystallographic transformations. Atomic features such as atomic number, electronegativity, and covalent radius were included. For explainable feature extraction and to gain insights into the model predictions, SHAP (SHapley Additive exPlanations) analysis was utilized[15]. To assess whether the model truly learns meaningful structure–property relationships rather than memorizing spurious patterns, we perform *y*-scrambling. Specifically, the target labels (*y*) are randomly shuffled while

keeping the input features (x) unchanged, thus breaking the original x–y relationship. The model is then retrained under identical hyperparameters on the scrambled dataset, and this process is repeated multiple times.

Calculation of the Correlation Coefficient

To quantitatively assess the linear relationship between the first principal component (PC1) scores and the HOMO-LUMO energy gaps of the same set of molecules, we perform a Pearson correlation analysis. Let $PC1_i$ denote the PC1 score of the ith molecule, and Gap_i represent the corresponding HOMO-LUMO energy gap. The Pearson correlation coefficient rrr is calculated using the following formula:

$$\boldsymbol{r} = \frac{\sum_{i=1}^{M}(PC1_i - \overline{PC1})(Gap_i - \overline{Gap})}{\sqrt{\sum_{i=1}^{M}(PC1_i - \overline{PC1})^2}\sqrt{\sum_{i=1}^{M}(Gap_i - \overline{Gap})^2}}$$

where: $\overline{PC1}$is the mean of the PC1 scores. $\overline{Gap}$ is the mean of the HOMO-LUMO energy gaps. M is the total number of molecules in the dataset.

Ionic Dynamics Testing of Polymer Electrolytes

Polymer electrolyte membranes were prepared by cutting them into discs with a diameter of 19 mm. These discs were then assembled into 2032 coin cells by sandwiching them between stainless steel and lithium metal electrodes for cyclic voltammetry (CV) and linear sweep voltammetry (LSV) measurements. For CV tests, the voltage was swept from -1 V to 4.9 V at a scan rate of 1 mV/s. LSV measurements were conducted by scanning the voltage from the open-circuit potential up to 6 V at a scan rate of 10 mV/s. To evaluate lithium-ion conductivity, symmetric Li||Li cells were constructed by placing the electrolyte discs between two lithium metal electrodes. Electrochemical impedance spectroscopy (EIS) was performed over a frequency range from 1 MHz to 1 Hz with a perturbation amplitude of 10 mV. The ionic conductivity (σ) was calculated using the equation:

$$\sigma = \frac{L}{R \cdot S}$$

where L is the thickness of the solid polymer electrolyte (SPE) membrane, R is the bulk resistance obtained from the EIS measurements, and S is the contact area between the stainless steel electrode

and the polymer electrolyte membrane. Lithium-ion transference numbers (t_{Li^+}) were determined using symmetric cells under a DC polarization of 10 mV. The transference number was calculated using the Bruce-Vincent-Evans equation[16]

$$t_{Li^+} = \frac{I_s \cdot R_{bs} \cdot (\Delta V - I_0 \cdot R_0)}{I_0 \cdot R_{b0} \cdot (\Delta V - I_s \cdot R_s)}$$

In this equation, ΔV is the applied DC voltage (10 mV), I_0 and I_s are the initial and steady-state currents, R_0 and R_s are the initial and steady-state charge-transfer resistances, and R_{b0} and R_{bs} are the initial and steady-state bulk resistances of the polymer electrolytes. The activation energy (E_a) for lithium-ion conduction was calculated using the Arrhenius equation[17]:

$$\sigma = A \cdot exp\left(\frac{-E_a}{k_b T}\right)$$

where A is the pre-exponential factor, k_b is the Boltzmann constant, and T is the absolute temperature.

Fabrication of PVDF-HFP@TFDMA and PVDF-HFP@TFOMA SPEs Membranes

Following cathode preparation, polymer electrolyte membranes were fabricated as follows. For PVDF-HFP@TFDMA membranes, PVDF-HFP (Mw ~ 400,000, Sigma-Aldrich), LiTFSI, and TFDMA were combined at a mass ratio of 1:1:10. First, PVDF-HFP and TFDMA were mixed and stirred at 80 °C for 6 hours until the PVDF-HFP fully dissolved. Subsequently, LiTFSI was added, and the mixture was stirred for an additional 2 hours to ensure complete dissolution of the lithium salt. The resulting solution was then cast onto a clean glass substrate using a doctor blade, and after the surface solvent had fully evaporated, the resulting membrane was transferred to a vacuum oven at 60 °C and dried for 30 minutes. All sample preparation steps were conducted in an argon-filled glovebox to maintain an inert environment. PVDF-HFP@TFOMA membranes were prepared following a similar procedure, with TFDMA replaced by TFOMA under analogous conditions.

Assembly and Performance Testing of Solid-State Lithium Metal Full Cells

Cathodes were fabricated using a conventional slurry-coating method. Active materials such as $LiFePO_4$ (LFP) and NCM91 were mixed with Ketjenblack carbon and polyvinylidene fluoride (PVDF) binder in N-methyl-2-pyrrolidone (NMP) solvent at a weight ratio of 8:1:1. The slurry was cast onto carbon-coated aluminum foil and dried under vacuum at 80 °C for 12 hours, achieving an active material loading of approximately 2.5–3 mg cm^{-2}. For cathodes with higher mass loading, LFP,

Ketjenblack carbon, PVDF, and LiTFSI were mixed in a weight ratio of 8:1:1:1. The cathode sheets were then punched into discs with a diameter of 14 mm for assembly into CR2032-type coin cells.

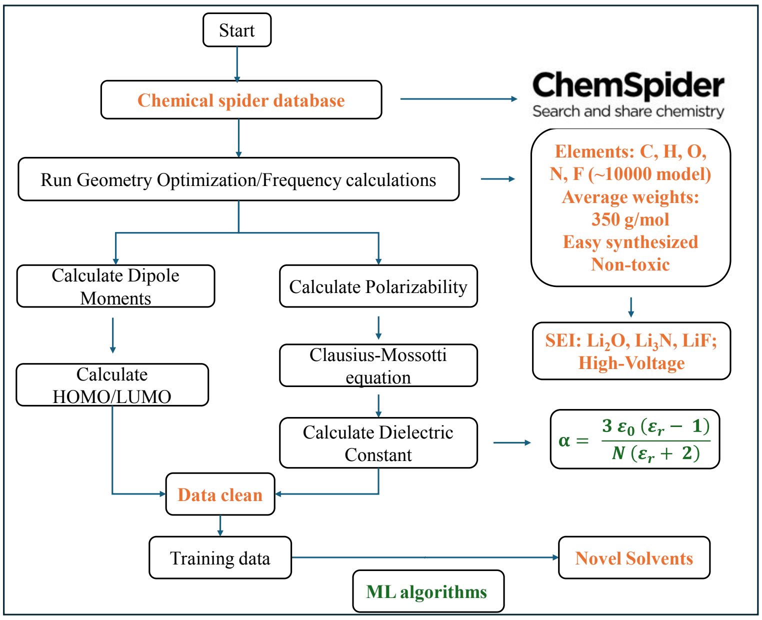

Figure S1. Workflow diagram for high-throughput screening and machine learning prediction of novel solvents using the Chemical Spider database. The relative dielectric constant (ε) is calculated using $\varepsilon = 3\varepsilon_0(\alpha_r - 1) / N(\alpha_r + 2)$, where ε_0 is the vacuum permittivity (8.854×10^{-12} F/m). αr is the molecular polarizability obtained from DFT calculations, and N is the number of molecules per unit volume.

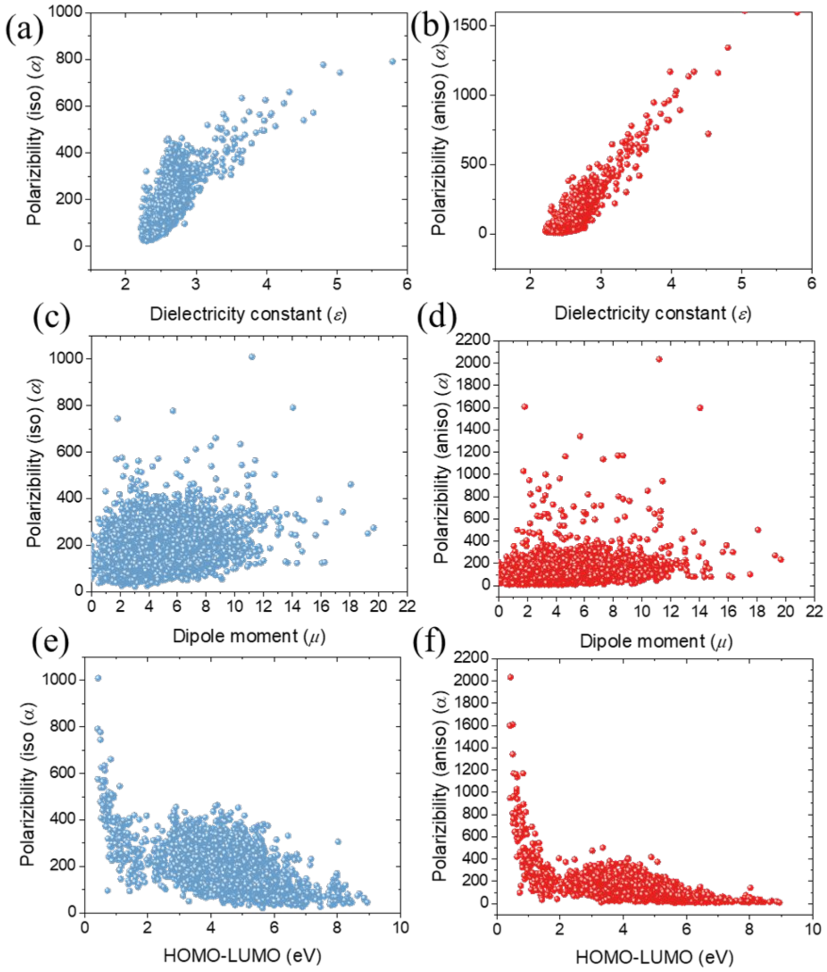

Figure S2. Relationships between (a, b) molecular polarizability (α) and dielectric constant (ε), (c, d) dipole moment (μ), and (e, f) HOMO-LUMO gap. Panels (a, c, e) show isotropic molecular polarizability, while panels (b, d, f) display anisotropic molecular polarizability.

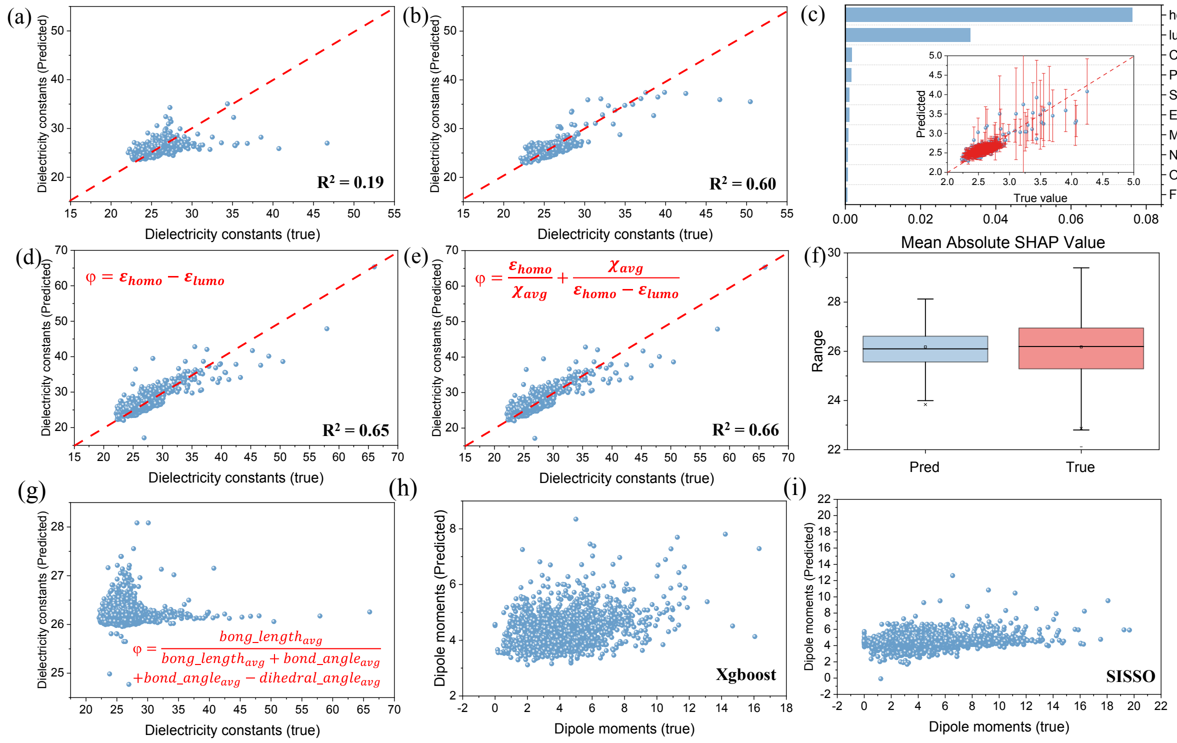

Figure S3. (a, b) Test results considering (a) and not considering (b) geometric structural factors based on the XGBoost algorithm; (c) (c) SHAP feature importance analysis from the training results, with the inset showing the true vs predicted dielectric constants with uncertainty using XGBoost. (d) and not considering (e) electronegativity factors based on the SISSO algorithm; (f) box plots comparing predicted and actual values. (g) dielectric constant prediction results using the SISSO algorithm without considering HOMO-LUMO features. (h,i) Dipole moment prediction results using machine learning algorithms: (h) Results based on the XGBoost algorithm and (i) SISSO algorithm.

Table S1. Pearson correlation coefficients (and corresponding *p*-values) between the model-derived features and various material properties across three different datasets

Feature-Property	Dielectricity		Dipole moments		HOMO-LUMO	
	Correlation	*p*-value	Correlation	*p*-value	Correlation	*p*-value
**Predictions *vs* true**	0.128	$9.83e^{-26}$	0.805	0.0	-0.198	$4.08e^{-60}$
**Predictions vs HOMO**	-0.568	0.0	-0.158	$\sim 9.00e^{-39}$	0.941	0.0
**Predictions vs LUMO**	0.937	0.0	0.078	$\sim 1.93e^{-10}$	-0.569	0.0
**PC1 vs dipole_moments**	-0.506	0.0	-0.506	0.0	-0.506	0.0
**PC1 vs HOMO-LUMO**	-0.0465	$\sim 1.44e^{-4}$	-0.0465	$\sim 1.44e^{-4}$	-0.0465	$\sim 1.44e^{-4}$
**PC2 vs dipole_moments**	0.306	$\sim 2.75e^{-145}$	0.306	$\sim 2.75e^{-145}$	0.306	$\sim 2.75e^{-145}$

**PC2 vs HOMO-LUMO**	-0.0422	~5.62e^{-4}	-0.0422	~5.62e^{-4}	-0.0422	~5.62e^{-4}
**t-SNE_x vs dipole_moments**	-0.496	0.0	-0.496	0.0	-0.496	0.0
**t-SNE_x vs HOMO-LUMO**	-0.0146	~0.231	-0.0146	~0.231	-0.0146	~0.231
**t-SNE_y vs dipole_moments**	0.213	~2.83e^{-69}	0.213	~2.83e^{-69}	0.213	~2.83e^{-69}
**t-SNE_y vs HOMO-LUMO**	-0.0238	~0.0515	-0.0238	~0.0515	-0.0238	~0.0515

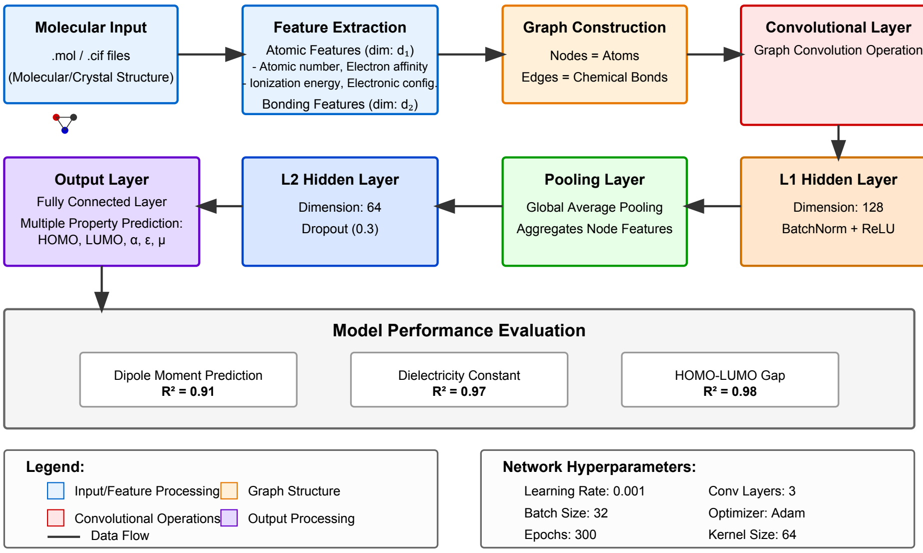

Figure S4. Improved crystal graph convolutional neural network (CGCNN) architecture

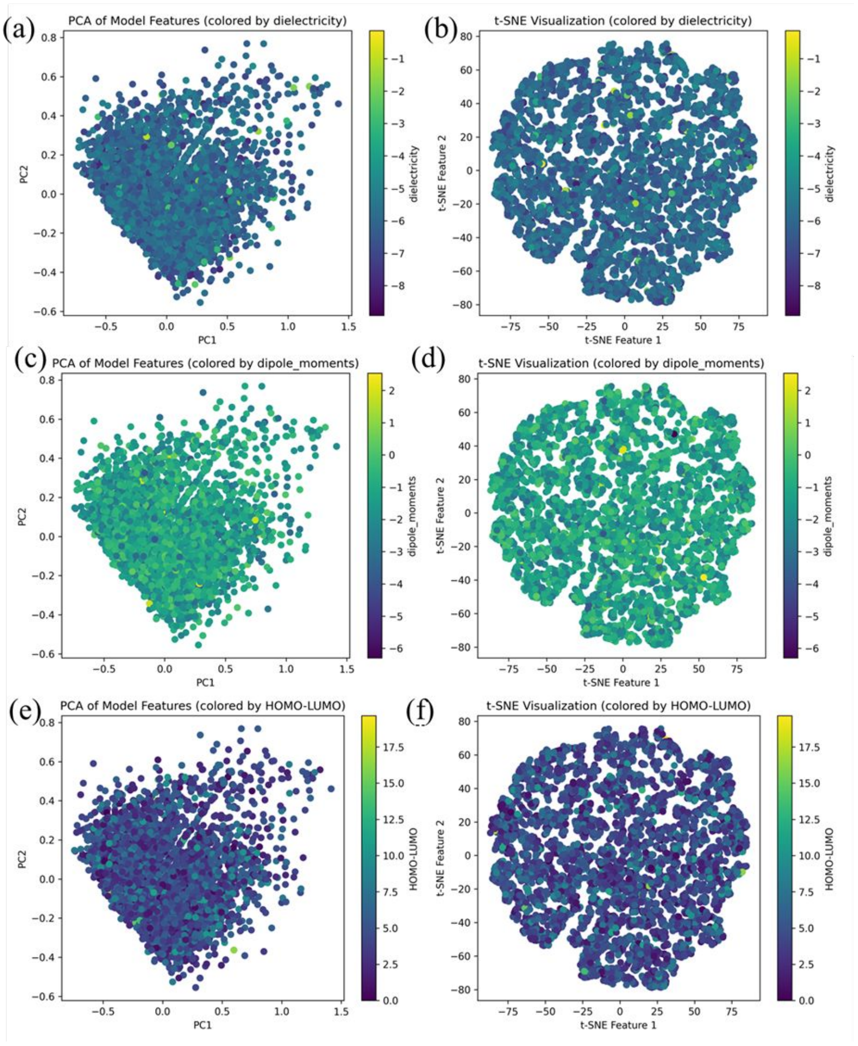

Figure S5. Feature space visualizations using principal component analysis (PCA, left) and t-distributed stochastic neighbor embedding (t-SNE, right) colored by (a,b) dielectricity (-8 to -1), (c,d) dipole moments (-6 to 2), and (e,f) HOMO-LUMO gaps (0 to 17.5). PCA projections show the first two principal components, while t-SNE reveals local structure of the high-dimensional features.

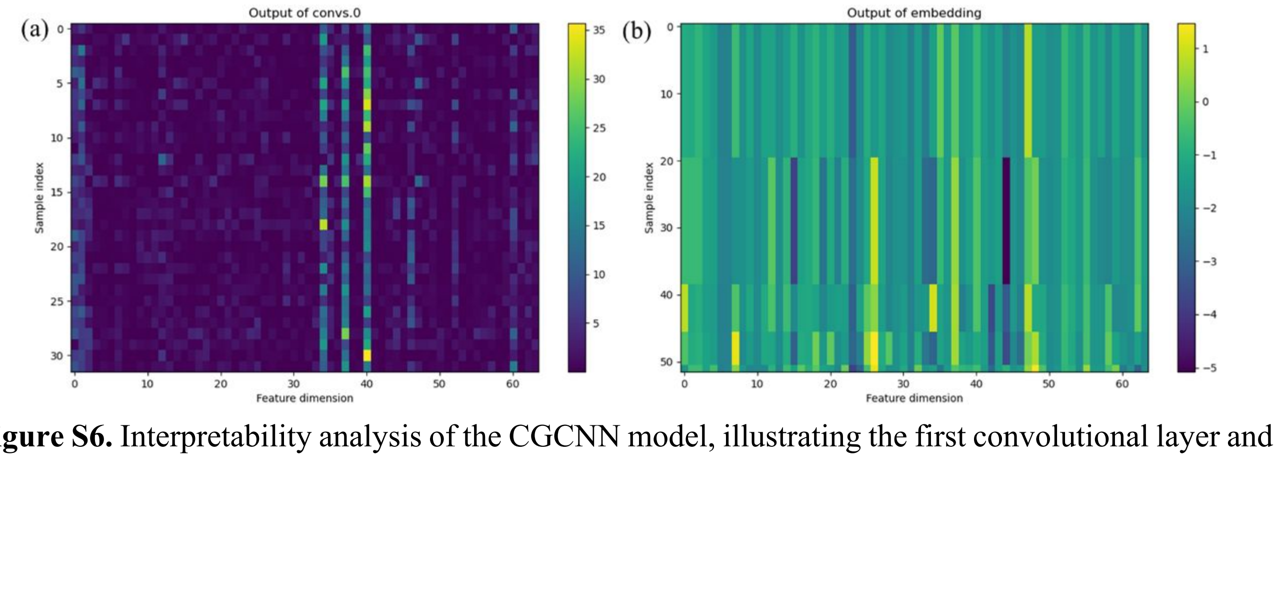

Figure S6. Interpretability analysis of the CGCNN model, illustrating the first convolutional layer and

the first hidden layer from the HOMO-LUMO training model.

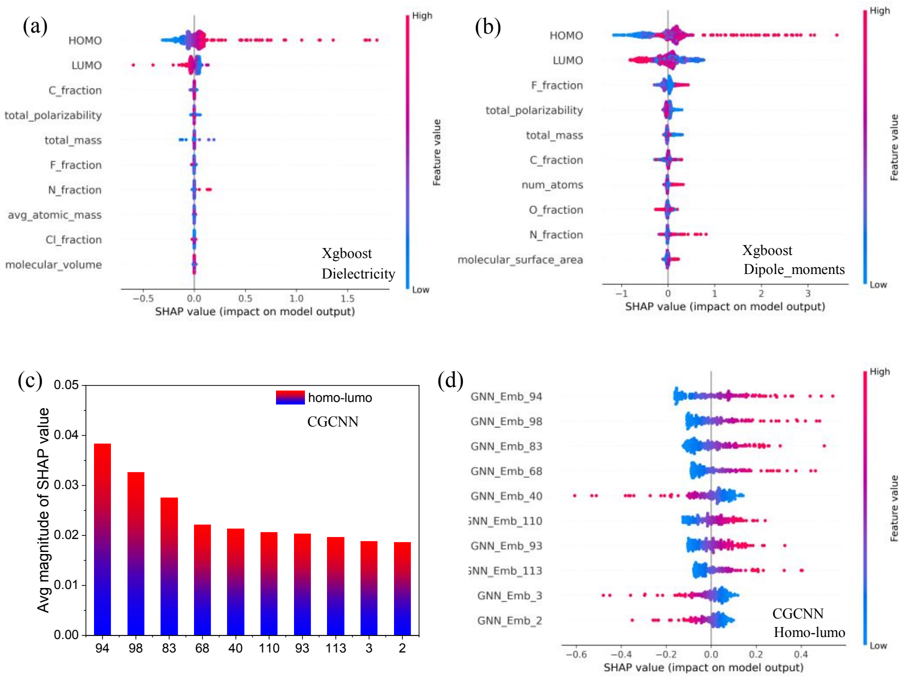

Figure S7. (a, b) SHAP analysis of the XGBoost-based models for dielectricity (a) and dipole_moments (b). (c, d) Feature importance analysis (c) and the corresponding SHAP distribution (d) for the homo–Lumo training model based on the CGCNN algorithm.

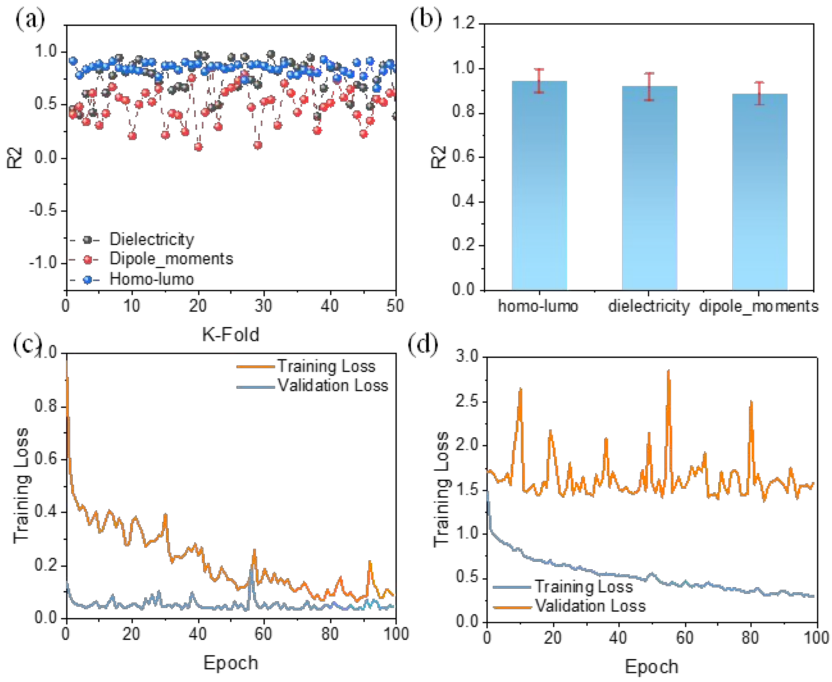

Figure S8. (a, b) R^2 scores for the CGCNN-based predictions of Dielectricity, Dipole_moments, and HOMO–LUMO across 50-fold cross-validation (a) and multiple random seeds (b). (c, d) Training loss curves over 100 epochs for the HOMO–LUMO model without (c) and with (d) y-sampling.

Table S2. Basic physical and chemical properties of common lithium-ion battery solvents.

Solvent	Full Name	Polarity	Dielectric Constant	Dipole Moment	Donor Number	HOMO (eV)	LUMO (eV)	Gap (eV)	Function in Electrolyte
**EC**	Ethylene Carbonate	High	89.8	4.81	16.4	-7.9	-0.97	6.93	Primary solvent high dielectric constant crucial for SEI formation
**PC**	Propylene Carbonate	High	64.9	4.94	15.1	-7.5	-0.85	6.65	High dielectric constant wide temperature range
**DMC**	Dimethyl Carbonate	Low	3.1	0.76	15.1	-7.2	-0.75	6.45	Low viscosity co-solvent improves conductivity
**DEC**	Diethyl Carbonate	Low	2.8	0.96	16	-7	-0.65	6.35	Low viscosity co-solvent improves wettability
**EMC**	Ethyl Methyl Carbonate	Low	2.9	0.89	15.5	-7.1	-0.7	6.4	Balance between DMC and DEC properties
**FEC**	Fluoroethylene Carbonate	High	78.4	4.91	14.6	-8.1	-1.05	7.05	SEI film forming additive
**DMSO**	Dimethyl Sulfoxide	High	46.7	3.96	29.8	-6.8	-0.55	6.25	High solvating ability
**NMP**	N-Methyl-2-pyrrolidone	High	32.2	4.09	27.3	-6.5	-0.45	6.05	Electrode manufacturing
**DMF**	N N-Dimethylformamide	High	36.7	3.82	26.6	-6.7	-0.5	6.2	High solvating power
**ACN**	Acetonitrile	Medium	37.5	3.92	14.1	-8.2	-1.1	7.1	High conductivity
**THF**	Tetrahydrofuran	Low	7.4	1.75	20	-6.9	-0.6	6.3	Good solvating ability
**DOL**	1,3-Dioxolane	Low	7.1	1.47	18	-7	-0.65	6.35	Li-S batteries flexible SEI
**TMP**	Trimethyl Phosphate	Medium	21	2.82	23	-7.3	-0.8	6.5	Flame retardant
**TEGDME**	Tetraethylene Glycol Dimethyl Ether	Medium	7.9	2.31	16.6	-6.8	-0.55	6.25	High chemical stability used in Li-air batteries

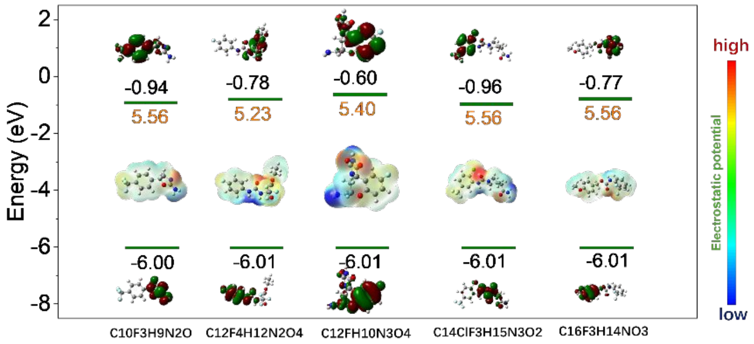

Figure S9. HOMO-LUMO results for the last five solvents obtained based on the screening criteria: $C_{10}F_3H_9N_2O$, $C_{12}F_4H_{12}N_2O_4$, $C_{12}FH_{10}N_3O_4$, $C_{14}F_3H_{15}N_3O_2$, and $C_{16}F_3H_{14}NO_3$.

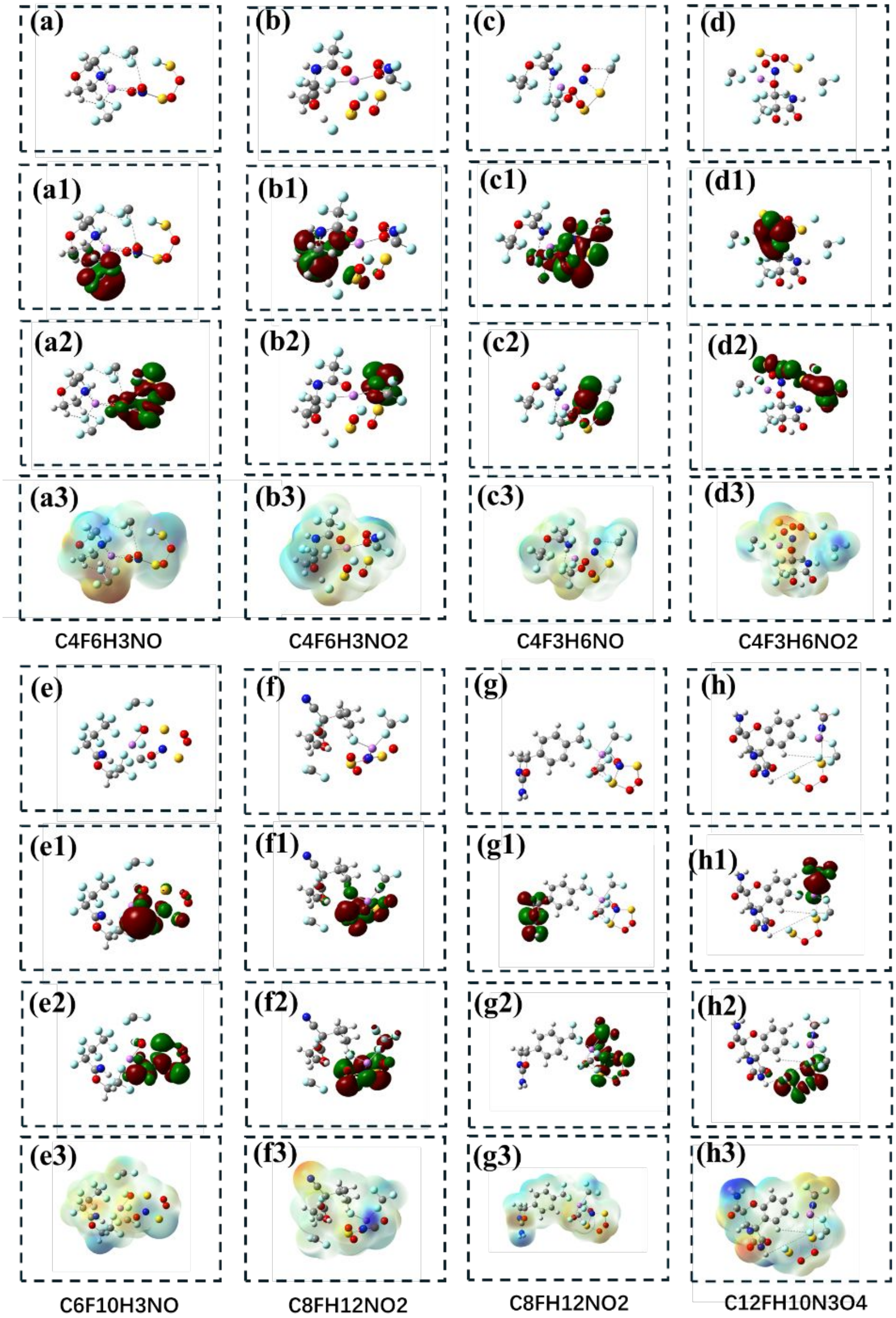

Figure S10. Optimized adsorption models of the top eight solvents with lithium salt (LiTFSI): From top to bottom—(a–a$_3$) $C_4F_6H_3NO$, (b–b$_3$) $C_4F_6H_3NO_2$, (c–c$_3$) $C_4F_3H_6NO$, (d–d$_3$) $C_4F_3H_6NO_2$, (e–e$_3$) $C_6F_{10}H_3NO$, (f–f$_3$) $C_8FH_{12}NO_2$, (g–g$_3$) another isomer of $C_8FH_{12}NO_2$, and (h–h$_3$) $C_{12}FH_{10}N_3O_4$. For each solvent: From left to right—(a–h) optimized geometric structures, (a_1–h_1) HOMO isosurfaces, (a_2–h_2) LUMO isosurfaces, and (a_3–h_3) electrostatic potential maps.

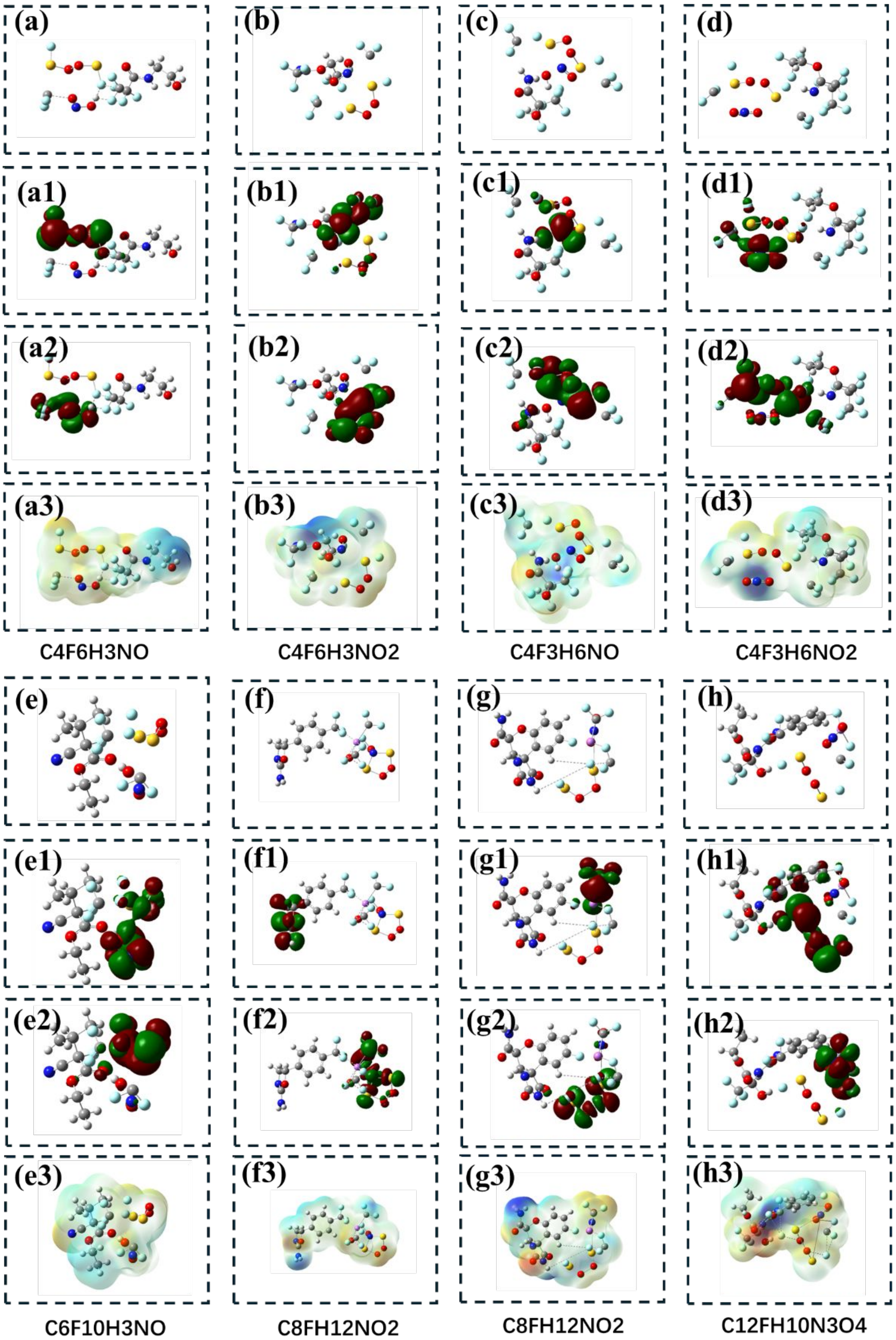

Figure S11. Optimized adsorption models of the top eight solvents with HTFSI: From top to bottom—(a–a_3) $C_4F_6H_3NO$, (b–b_3) $C_4F_6H_3NO_2$, (c–c_3) $C_4F_3H_6NO$, (d–d_3) $C_4F_3H_6NO_2$, (e–e_3) $C_6F_{10}H_3NO$, (f–f_3) $C_8FH_{12}NO_2$, (g–g_3) another isomer of $C_8FH_{12}NO_2$, and (h–h_3) $C_{12}FH_{10}N_3O_4$. For each solvent: From left to right—(a–h) optimized geometric structures, (a_1–h_1) HOMO isosurfaces, (a_2–h_2) LUMO isosurfaces, and (a_3–h_3) electrostatic potential maps.

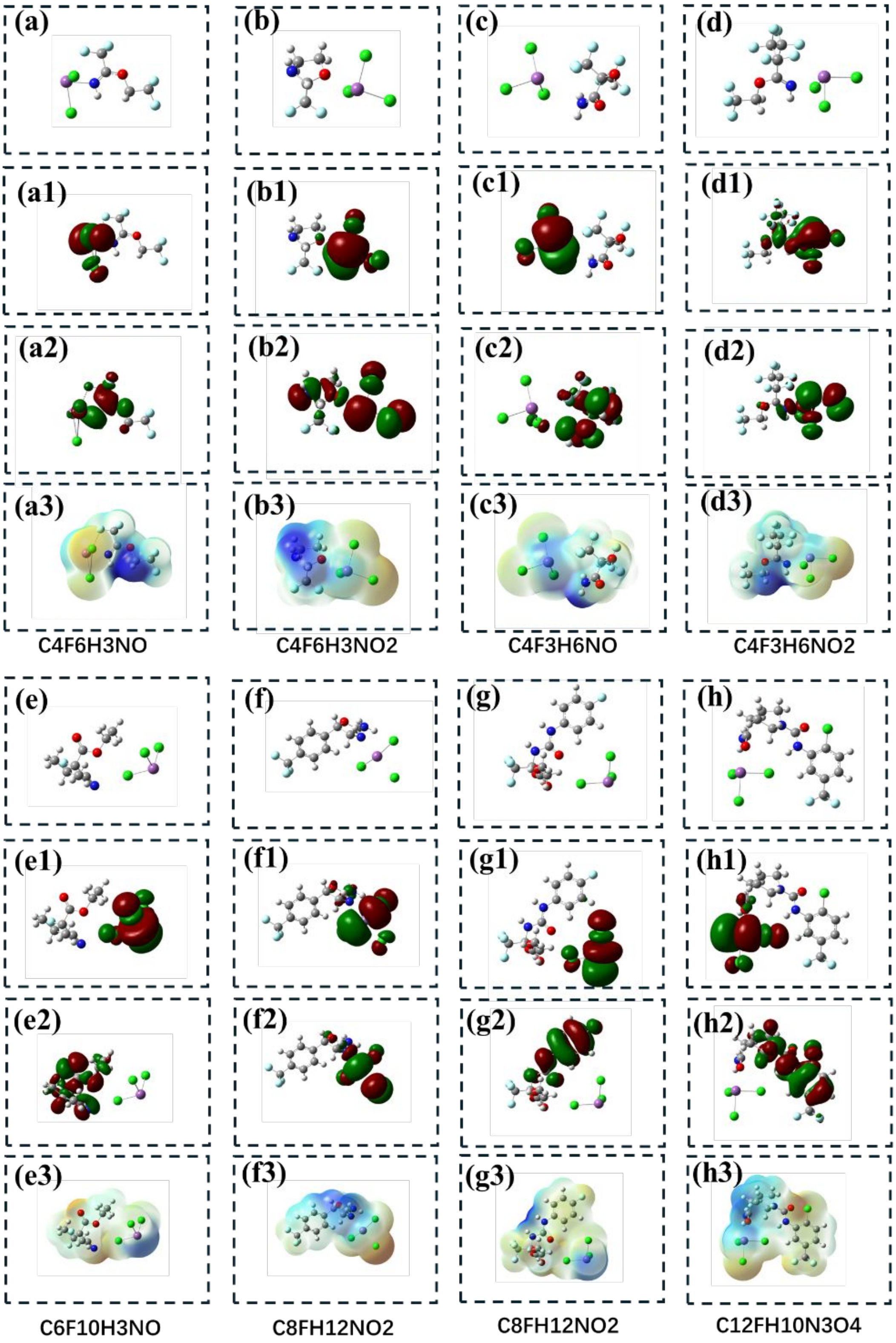

Figure S12. Optimized adsorption models of the top eight solvents with typical lewis acids ($SbCl_5$): From top to bottom—(a–a_3) $C_4F_6H_3NO$, (b–b_3) $C_4F_6H_3NO_2$, (c–c_3) $C_4F_3H_6NO$, (d–d_3) $C_4F_3H_6NO_2$, (e–e_3) $C_6F_{10}H_3NO$, (f–f_3) $C_8FH_{12}NO_2$, (g–g_3) another isomer of $C_8FH_{12}NO_2$, and (h–h_3) $C_{12}FH_{10}N_3O_4$. For each solvent: From left to right—(a–h) optimized geometric structures, (a_1–h_1) HOMO isosurfaces, (a_2–h_2) LUMO isosurfaces, and (a_3–h_3) electrostatic potential maps.

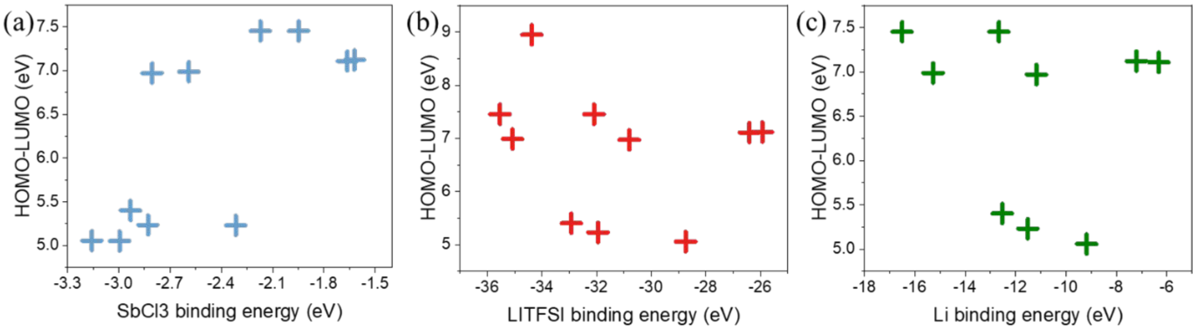

Figure S13. Relationships between adsorption energies and electronic properties: Adsorption energies of (a) $SbCl_5$, (b) LiTFSI, and (c) Li plotted against the HOMO-LUMO gap.

Table S3. Comparison of molecular properties predicted by DFT calculations and CGCNN model for selected molecules, including Dielectricity, Dipole moments and HOMO-LUMO gaps,

**Model**	**Dielectricity**		**Dipole_Moments**		**HOMO-LUMO**	
	DFT	CGCNN	DFT	CGCNN	DFT	CGCNN
C4F3H6NO2_1	23.5	23.3	4.96	4.21	6.63	6.01
C4F3H6NO2_2	23.3	23.4	1.50	1.76	7.45	6.91
C4F3H6NO_1	23.6	23.5	2.53	2.69	8.95	8.88
C4F3H6NO_2	23.5	23.5	3.20	4.09	8.46	8.52

Table S4. Physical properties of the top five solvents and two novel predicted solvents based on the screening criteria.

**Solvents**	**Dielectric constant (*ε*)**	**Dipole moment (*μ*)**	**homo-lumo (eV)**
**C4F6H3NO**	22.62	3.62	6.99
**C4F6H3NO2**	22.38	3.72	7.45
**C6F10H3NO**	22.39	3.54	6.97
**C6FH8NO2**	24.99	3.76	7.12
**C8FH12NO2**	25.40	3.55	7.11
**C4F3H6NO2**	**23.60**	**3.53**	**7.45**

**C4F3H6NO**	**23.31**	**3.50**	**8.95**

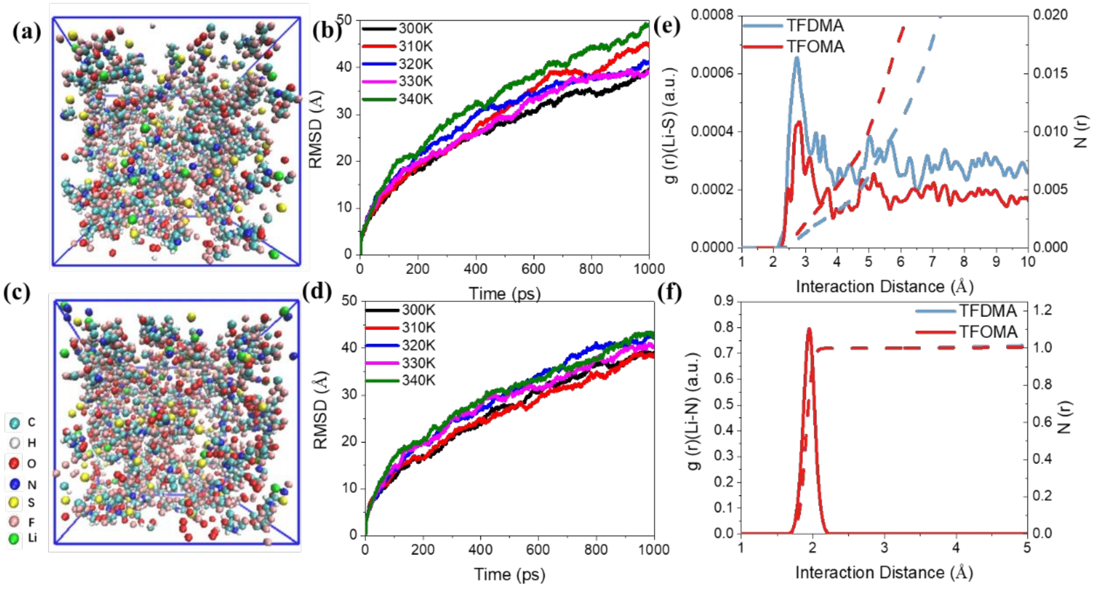

Figure S14. Molecular dynamics simulation results for solvent-LiTFSI mixtures: (a, b) Mixed model of TFDMA with LiTFSI (a) and corresponding RMSD plots of Li atoms at temperatures of 300 K, 310 K, 320 K, 330 K, and 340 K (b); (c, d) Mixed model of TFOMA with LiTFSI (c) and corresponding RMSD plots (d); (e, f) Radial distribution functions of Li–S atomic pairs (e) and Li–N atomic pairs (f) in TFDMA and TFOMA systems.

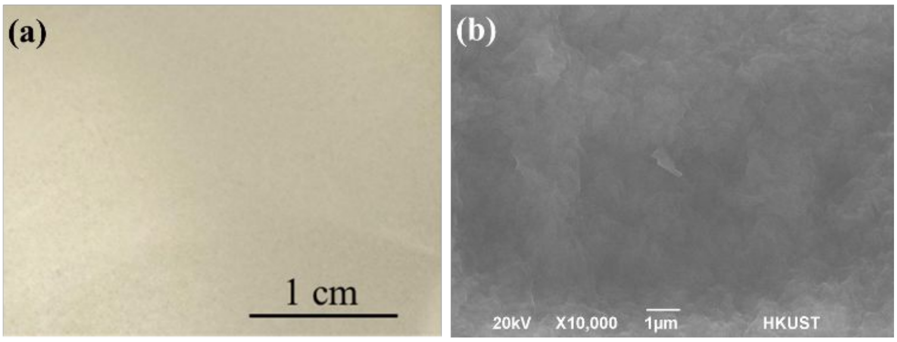

Figure S15. (a, b) Photographic image (a) and SEM image (b) of the PVDF-HFP@TFOMA electrolyte membrane.

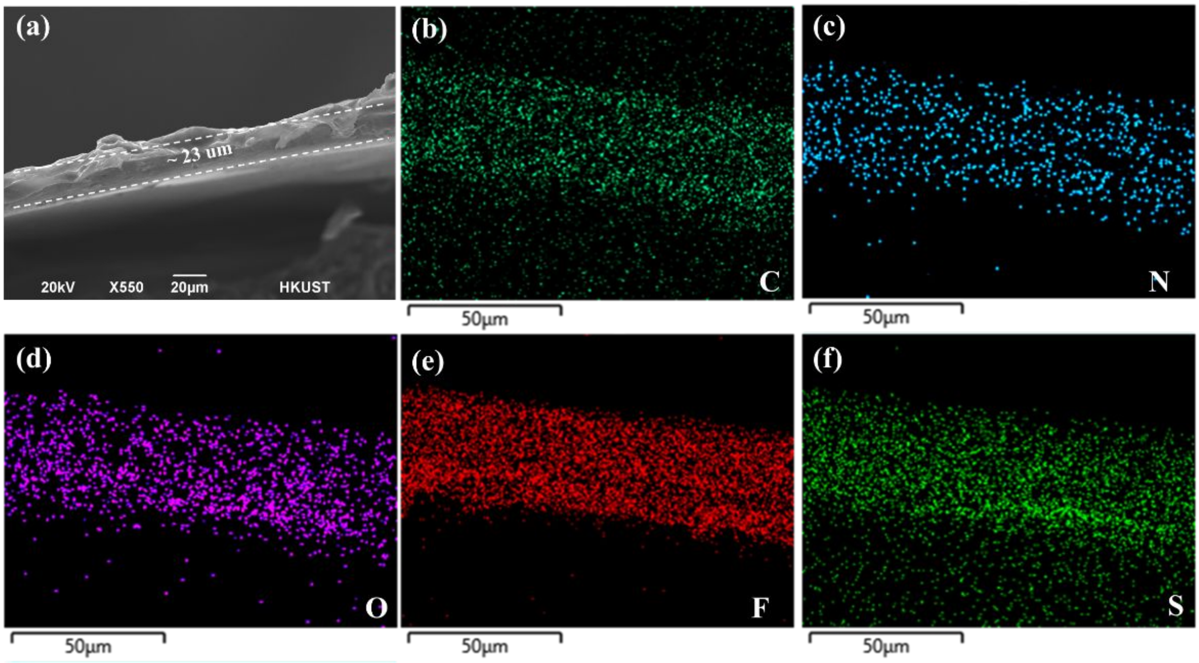

Figure S16. (a) Cross-sectional SEM image of the PVDF-HFP@TFOMA electrolyte membrane, and corresponding elemental mapping for (b) carbon (C), (c) nitrogen (N), (d) oxygen (O), (e) fluorine (F), and (f) sulfur (S).

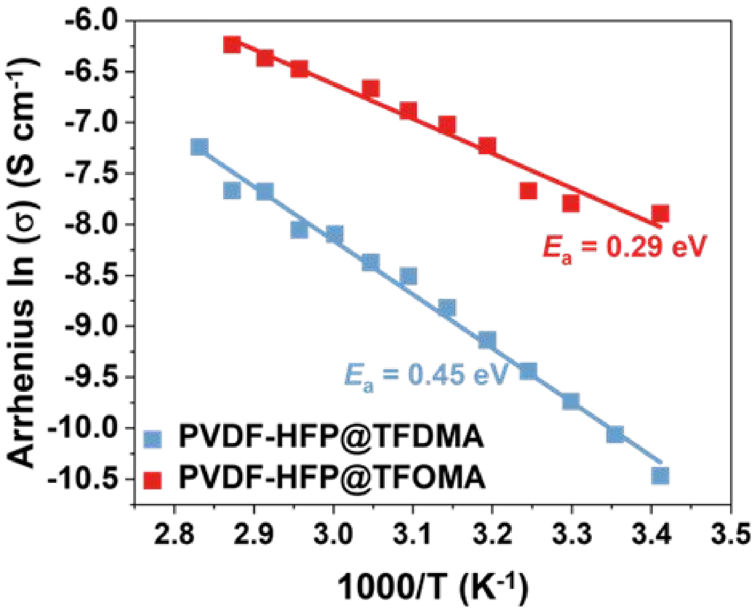

Figure S17. Activation energy measurements based on the Arrhenius equation, comparing lithium-ion activation energies for PVDF-HFP@TFDMA and PVDF-HFP@TFOMA electrolyte membranes.

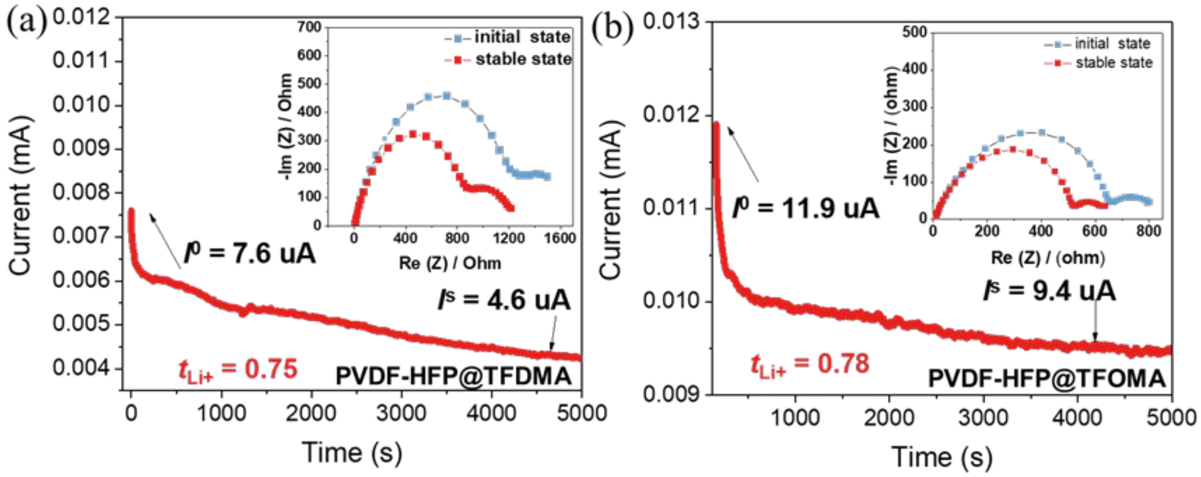

Figure S18. Lithium-ion transference number measurements for electrolyte membranes: (a) PVDF-HFP@TFDMA; (b) PVDF-HFP@TFOMA.

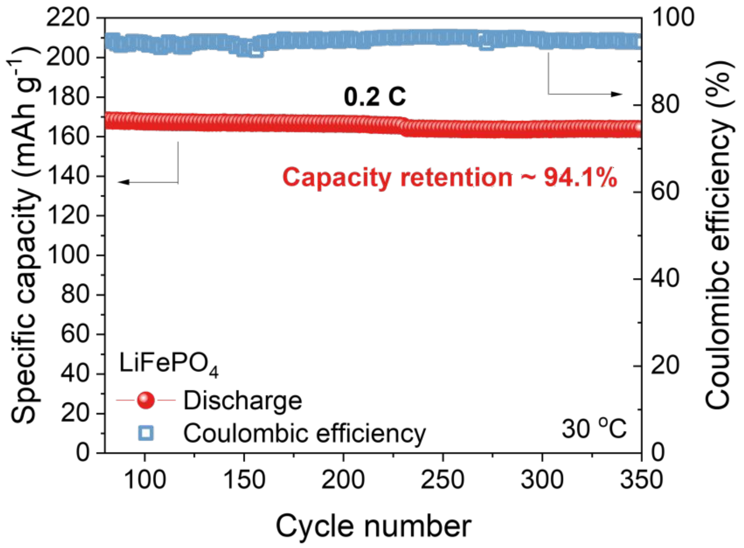

Figure S19. Electrochemical performance at 0.2 C (1 C = 174 mAh g^{-1}) (i) of $LiFePO_4$ cathode with PVDF-HFP@TFOMA solid electrolyte.

Table S5. Recent reports on electrochemical performance of PVDF-based polymer solid electrolytes with residual solvents.

Literature	Li salts	Polymer	Solvents	σ_{Li} (S cm^{-1}) (30 ^{o}C)	t_{Li+}	Performance
Angew. Chem. Int. Ed. **2021**, *60*, 24668	LiTFSI	PVDF@LATP	DMF	6.0×10^{-4}	0.58	NCM811: 160 mAh g^{-1}, 2 C, 1500 cycles, 61.4%
Adv. Funct. Mater. **2024**, 2409134	LiTFSI	PVDF-HFP	Adiponitrile (ADN)	5.1×10^{-4}	0.74	LFP: 146.8 mAh g^{-1} ,0.5 C, 300 cycles, 96.4%; NCM811: 173.4 mAh g^{-1}, 0.5 C, 100 cycles, 86.2%
Energy Storage Mater. **2024**, *71*, 103570	LiTFSI	PVDF-HFP	DMF	5.9×10^{-4}	0.48	LFP: 166.76 mAh g^{-1},1 C, 1200 cycles 82.83%
J. Am. Chem. Soc. **2023**, *145*, 25632	LiTFSI and LiDFOB	PVDF@LATP	NMP	2.2×10^{-3}	0.56	LFP: 140 mAh g^{-1}, 5 C, 2000 cycles, 80%;
Adv. Mater. **2024**, *36*, 2401549	LiTFSI	PVDF-HFP	FDMA	5.2×10^{-4}	0.43	LFP: 150 mAh g^{-1}, 1 C, 2500 cycles , 80.3% LCO: 141.5 mAh g^{-1}, 0.5 C, 800 cycles, 80.5%,
Nat. Commun. **2024**, *15*, 3914	LiFSI	PVDF-HFP	DMF	6.5×10^{-4}	0.71	Li|SPAN: 900 mAh g^{-1}, 1 C, 1000 cycles, 60%
Angew. Chem. Int. Ed. **2024**, *63*, e202401428	LiTFSI	PVDF	DMF	4.5×10^{-4}	0.47	NCM811: 165mAh g^{-1}, 1 C, 1130 cycles, 68.7%,
Energy Storage Mater. **2024**, *71*, 103570	LiTFSI	PVDF-HFP	DMF	5.9×10^{-4}	0.48	LFP: 166.76 mAh g^{-1}, 1 C, 1200 cycles, 82.83%.
Small Science **2023**, *3*, 2300017	LiFSI	PVDF-HFP	DMF	1.33×10^{-3}	0.54	LFP: 145 mAh g^{-1}, 0.4 C, 180 cycles, 94.5%.
Adv. Energy Mater. **2022**, *12*, 2200967	LiTFSI	PVDF-HFP	DMF	1.32×10^{-4}	0.47	NCM622: 173.1 mAh g^{-1}, 1 C, 300 cycles, 81.5%.
J. Energy Chem. **2023**, *81*, 613–622	LiTFSI	PVDF-HFP	NMP	7.4×10^{-4}	0.41	LFP: 120 mAh g^{-1}, 1 C, 600 cycles, 85.3%.
J. Energy Storage **2023**, *68*, 107810	LiODFB	PVDF-HFP	NMP	7.4×10^{-4}	0.65	LFP: 152.0 mAh g^{-1}, 1 C, 150 cycles, 100% NCM811: 158.9 mAh g^{-1}, 0.1 C, 100 cycles, 85.8%
**This work**	**LiTFSI**	**PVDF-HFP**	**C4F3H6NO2 (TFOMA)**	$\mathbf{5.5 \times 10^{-4}}$	**0.78**	**LFP: 150 mAh g^{-1}, 2 C, 500 cycles, 86.7%; NCM91: 170 mAh g^{-1}, 1 C, 150 cycles, 98.7%**

Table S6 Machine-learning comparison of electrolytes based on batteries

Literatures	Systems	ML-Algorithms	Validation Method	Physical Explainability?	Closed-Loop?
**J. Am. Chem. Soc. 2023, 145, 23764**	Liquid electrolytes	Random Forest (RF) combined with SHAP analysis	DFT calculations	√	×
**PNAS 2023, 120, e2214357120**	Liquid electrolytes	Linear Regression (LR), Random Forest (RF), and Bagging Regression (BR)	Experiments	×	√
**J. Phys. Chem. C 2023, 127, 14636**	Na–ion SSEs	Random Forest (RF) with SHAP analysis for regression	Experiments	×	×
**Science Bulletin 66 (2021) 1401**	Li–ion SSEs	Partial Least Squares (PLS) Regression using HECS descriptors	DFT calculations	×	×
**ACS Cent. Sci. 2018, 4, 996**	Li–ion SSEs	Partial Least Squares (PLS) Regression using HECS descriptors	DFT calculations	×	×
**ACS Appl. Mater. Interfaces 2021, 13, 42590**	Li–ion SSEs	Random Forest (RF) enhanced via an active learning framework	DFT calculations	×	×
**Angew. Chem. Int. Ed. 2025, 64, e202411437**	Li–ion SSEs	Stacking ensemble model integrating Linear Regression (LR), Gradient Boosting Regressor (GB), and Extra Trees Regressor (ET) with SHAP analysis	DFT+MD+exp	√	√
**ACS Cent. Sci. 2023, 9, 206–216**	Li–ion SSEs	Message Passing Neural Network (MPNN) with an Arrhenius-informed readout layer (ChemArr)	exp	√	√
**Nat. Commun. 2023, 14:2789**	Li–ion SSEs	Ensemble workflow combining supervised methods (SVM, RF, XGBoost) and Graph Convolutional Neural Network (GCNN) for classification and regression	DFT+EXP	√	√
This work	Li–ion SSEs	GNN, Xgboost, SISSO	DFT+MD+exp	√	√

References

(1) Böttcher, C. J. F. CHAPTER III - POLARIZATION AND ENERGY. In *Theory of Electric Polarization (Second Edition)*; Böttcher, C. J. F., Ed.; Elsevier: Amsterdam, 1973; pp 91–127.

(2) AnonymousModern Quantum Chemistry: Introduction to Advanced Electronic Structure Theory (Dover Books on Chemistry): 9780486691862: Attila Szabo, Neil S. Ostlund. In .

(3) AnonymousGaussian 16, state-of-the-art capabilities for electronic structure modeling.

(4) AnonymousASE, an Atomic Simulation Environment.

(5) Pence, H. E.; Williams, A. ChemSpider: An Online Chemical Information Resource. *J. Chem. Educ.* **2010**, *87*, 1123–1124.

(6) Binkley, J. S.; Pople, J. A.; Hehre, W. J. Self-consistent molecular orbital methods. 21. Small split-valence basis sets for first-row elements. *J. Am. Chem. Soc.* **1980**, *102*, 939–947.

(7) Grimme, S.; Antony, J.; Ehrlich, S.; Krieg, H. A consistent and accurate ab initio parametrization of density functional dispersion correction (DFT-D) for the 94 elements H-Pu. *The Journal of Chemical Physics* **2010**, *132*, 154104.

(8) Theodorou, D. N.; Suter, U. W. Detailed molecular structure of a vinyl polymer glass. *Macromolecules* **1985**, *18*, 1467–1478.

(9) Sun, H. COMPASS: An ab Initio Force-Field Optimized for Condensed-Phase ApplicationsOverview with Details on Alkane and Benzene Compounds. *J. Phys. Chem. B* **1998**, *102*, 7338–7364.

(10) Posch, H. A.; Hoover, W. G.; Vesely, F. J. Canonical dynamics of the Nos\'e oscillator: Stability, order, and chaos. *Phys. Rev. A* **1986**, *33*, 4253–4265.

(11) Parrinello, M.; Rahman, A. Crystal Structure and Pair Potentials: A Molecular-Dynamics Study. *Phys. Rev. Lett.* **1980**, *45*, 1196–1199.

(12) AnonymousXGBoost(eXtreme Gradient Boosting) Documentation. *https://xgboost.readthedocs.io/en/stable/index.html* (accessed Oct 31, 2024).

(13) Ouyang, R.; Curtarolo, S.; Ahmetcik, E.; Scheffler, M.; Ghiringhelli, L. M. SISSO: A compressed-sensing method for identifying the best low-dimensional descriptor in an immensity of offered candidates. *Phys. Rev. Mater.* **2018**, *2*, 083802.

(14) Xie, T.; Grossman, J. C. Crystal Graph Convolutional Neural Networks for an Accurate and Interpretable Prediction of Material Properties. *Phys. Rev. Lett.* **2018**, *120*, 145301.

(15) AnonymousSHAP (SHapley Additive exPlanations). *https://shap.readthedocs.io/en/latest/* (accessed Oct 31, 2024).

(16) Siekierski, M.; Bukat, M.; Ciosek, M.; Piszcz, M.; Mroczkowska-Szerszeń, M. Transference Number Determination in Poor-Dissociated Low Dielectric Constant Lithium and Protonic Electrolytes. *Polymers* **2021**, *13*, 895.

(17) Wan, H.; Xu, J.; Wang, C. Designing electrolytes and interphases for high-energy lithium batteries. *Nat Rev Chem* **2024**, *8*, 30–44.
